# Characterization of *Codonopsis pilosula* subsp. *tangshen* plastome and comparative analysis of *Codonopsis* species

**DOI:** 10.1371/journal.pone.0271813

**Published:** 2022-08-01

**Authors:** Jingwen Yue, Yang Ni, Mei Jiang, Haimei Chen, Pinghua Chen, Chang Liu

**Affiliations:** 1 Key Laboratory of Ministry of Education for Genetics, Breeding and Multiple Utilization of Crops, National Engineering Research Center of Sugarcane, College of Agriculture, Fujian Agriculture and Forestry University, Fuzhou, Fujian Province, P. R. China; 2 Key Laboratory of Bioactive Substances and Resource Utilization of Chinese Herbal Medicine from Ministry of Education, Engineering Research Center of Chinese Medicine Resources from Ministry of Education, Institute of Medicinal Plant Development, Chinese Academy of Medical Sciences, Peking Union Medical College, Beijing, P. R. China; G. B. Pant Institute of Himalayan Environment & Development, INDIA

## Abstract

*Codonopsis pilosula* subsp. *tangshen* is one of the most important medicinal herbs used in traditional Chinese medicine. Correct identification of materials from *C*. *pilosula* subsp. *tangshen* is critical to ensure the efficacy and safety of the associated medicines. Traditional DNA molecular markers could distinguish *Codonopsis* species well, so we need to develop super or specific molecular markers. In this study, we reported the plastome of *Codonopsis pilosula* subsp. *tangshen* (Oliv.) D.Y. Hong conducted phylogenomic and comparative analyses in the *Codonopsis* genus for the first time. The entire length of the *Codonopsis pilosula* subsp. *tangshen* plastome was 170,672 bp. There were 108 genes in the plastome, including 76 protein-coding genes, 28 transfer RNA (tRNA), and four ribosomal RNA (rRNA) genes. Comparative analysis indicated that *Codonopsis pilosula* subsp. *tangshen* had an unusual large inversion in the large single-copy (LSC) region compared with the other three *Codonopsis* species. And there were two dispersed repeat sequences at both ends of the inverted regions, which might mediate the generation of this inversion. We found five hypervariable regions among the four *Codonopsis* species. PCR amplification and Sanger sequencing experiments demonstrated that two hypervariable regions could distinguish three medicinal *Codonopsis* species. Results obtained from this study will support taxonomic classification, discrimination, and molecular evolutionary studies of *Codonopsis* species.

## 1. Introduction

The Campanulaceae family contains 50 genera and approximately 1046 species, mainly found in the north and south temperate regions [1]. *Codonopsis* is a genus of perennial herbs in the family Campanulaceae. It includes 42 species primarily distributed in East, Central, and South Asia. Among them, 40 are found in China [2].

Several *Codonopsis* species had been used as traditional Chinese medicines for one thousand years. Among them, the *Codonopsis pilosula* subsp. *tangshen* is one of the most used [3]. For example, Chuan Dan-Shen was the dried root of *C*. *pilosula* subsp. *tangshen* is produced in the Sichuan province of China. It has been regularly used to strengthen the spleen and tonify the lung, regulate blood sugar, lower blood pressure, strengthen the body’s immune system, and so on [4, 5]. Because of its high medicinal value and low price, Chuan Dan-Shen is sometimes used as a substitute for ginseng [6]. In addition, *C*. *pilosula* subsp. *tangshen* were used as food materials in southern China and Southeast Asia, such as tea, wine, soup, plaster, porridge, etc. [5].

Another species, *C*. *lanceolata* was used as a traditional medicinal plant and vegetable [7]. Previous research has reported that *C*. *lanceolata* has immune-modulatory, antimicrobial, antioxidant, and anti-inflammatory effects [8–10]. A third species, *C*. *tsinlingensis* has been used to lower blood pressure and treat poor appetite for a long time [2]. Materials from these species might be substituted for each other in traditional medicines, leading to potential problems in their efficacy and safety. As a result, understanding their phylogenetic relationship and developing molecular markers for these four species is urgently needed.

The application of DNA molecular markers in studying the genetic variation in *Codonopsis* has been reported [11] and showed that *Codonopsis* species were difficult to classify and discriminate using conventional markers. Previous studies have used nuclear internal transcribed spacer (nrITS) [12], microsatellite polymorphic loci [13], simple sequence repeats (SSRs) [14], inter simple sequence repeats (ISSR), random amplified polymorphic DNA (RAPD) [15], amplified fragment length polymorphism (AFLP), sequencing-based markers (SNP) to discriminate *Codonopsis* species. In particular, Hwang et al. used the genetic information from the plastomes of *Codonopsis lanceolata* and *Platycodon grandiflorus* for molecular marker development. Three chloroplast DNA (cp-DNA) based markers were developed from *ndh*F and *rpo*A genes using the linearity test of the Quantitative Real-time PCR (qRT-PCR) assay. And these three cp-DNA markers helped distinguish specific plant species between *C*. *lanceolata*, *P*. *grandiflorus*, and *Panax ginseng* in commercial mixed-flour products [16]. These studies suggest that the *Codonopsis* genus has a rich and complex species composition, highly similar morphological characteristics, dynamic evolutionary history, and extensive rearrangements of the plastomes during diversification. Therefore, high-resolution or specific molecular markers are needed to distinguish *Codonopsis* species [17–19].

Recent studies have compared the universal, super and specific DNA barcodes [20, 21]. The universal DNA barcodes include three chloroplast regions (*mat*K, *psb*A-*trn*H, and *rbc*L) and one nuclear region (ITS) [22, 23]. However, universal DNA barcodes do not work in the case of extremely closely related species or only slightly diverged “species” from a recent radiation event [24]. The super barcode includes a complete genome or parts of a genome containing enough information to discriminate between the species of interest [25]. The phylogenetic tree constructed based on complete plastomes has a higher supporting rate and discrimination power [26]. The specific barcode often uses hypervariable regions of the genome. One or a combination of several hypervariable regions can distinguish these more related species. However, super DNA barcodes are generally not recommended if commonly used universal or specific DNA barcodes can be accurately identified. It is a useful complement to current molecular identification [25]. Because a very large proportion of the plastome does not contribute much to species discrimination, the most variable regions could substitute the whole genome [20].

Among the 42 *Codonopsis* species, the complete plastomes of only three species have been published, including *Codonopsis lanceolata* (MH018574.1) [27] and *Codonopsis minima* (NC_036311.1) [28], and *Codonopsis tsinlingensis* (MN122102.1) [29]. In the current study, we sequenced the complete plastomes of *Codonopsis pilosula* subsp. *tangshen*, to identify super or specific barcode for the discrimination of closely related medicinal *Codonopsis* species We characterized the genomic features. Then we compared the plastomes from the four *Codonopsis* species. Lastly, we developed and validated a set of molecular markers to distinguish the four species. The results obtained from this study laid a solid foundation for future taxonomic classification and marker development studies for *Codonopsis* species. In the following text, *Codonopsis pilosula* subsp. *tangshen* and “tangshen” are interchangeable for the sake of easy reading.

## 2. Materials and methods

### 2.1 Plant material, DNA extraction, and sequencing

To obtain the complete plastome, we collected the fresh leaves of a young tangshen plant from the Huazhong Medicinal Botanical Garden (109°76’ E, 30°18’ N), Enshi, Hubei, China. To validate molecular markers of *Codonopsis* species, we collected fresh leaves of tangshen, *C*. *lanceolata*, and *C*. *tsinlingensis* from the Huazhong Medicinal Botanical Garden, Qichun Country, and the Qinling mountain, respectively. The detailed sample information used for sequencing and molecular marker validation is in the S1 Table in S1 File. All samples were collected with permission from the authorities.

Then we extracted the genomic DNA with the plant genomic DNA kit (Tiangen Biotech, China). The purity of total DNA was assessed by 1.0% agarose gel electrophoresis. And the concentrations were measured using a Nanodrop Spectrophotometer 2000 (Thermo Fisher Scientific Inc., Waltham, MA, USA). We used the library preparation kit (New England BioLabs, America) to construct the DNA library with 1ug DNA. For paired-end library construction, the total DNA was sheared into fragments at approximately 500 bp long. Finally, we sequenced the genomic DNA with a Hiseq 2500 platform (Illumina, San Diego, CA). The remaining sample and DNA were stored in the Institute of Medicinal Plant Development (IMPLAD, accession number: Implad201808044).

### 2.2 Genome assembly and annotation

After obtaining the raw data, we removed the low-quality sequences using Trimmomatic software [30] to get clean data. These low-quality sequences meet the following conditions: (1) having the adaptor sequences; (2) the sequences with more than 50% bases having quality values of Q < 19; and (3) with more than 5% bases being "N." With the development of next-generation sequencing technologies, generating organelle genome assemblies from whole-genome sequencing (WGS) data would be the most accurate and labor-saving method. The plastome of tangshen was de novo assembled using NOVOplasty (v4.0) [31] with the parameter "-t 15, -R 30". We validated the correctness of the assembly by mapping all raw reads to the assembly using BWA [32] with the default settings. The annotation of the plastome was conducted initially using the CPGAVAS2 [33] webserver. The annotation problems were edited by Apollo [34] manually. Then we updated the annotation results by using the "UpdateAnno" module in CPGAVAS2. The cis-splicing and trans-splicing genes of the tangshen plastome were created using CPGview-RSG (http://www.herbalgenomics.org/cpgview). The content of GC was calculated using Editseq from the DNASTAR Lasergene package (v9) [35]. Finally, we submitted the genome sequence and annotations to GenBank and obtained the accession number MW415426.

### 2.3 Repeat and IR regions boundary analysis

Microsatellites are repeating DNA sequences consisting of 1–6 nucleotides (tandem arrays). It is commonly found in the genomes of all prokaryotes and eukaryotes and is called motifs [36]. The microsatellite sequence was analyzed using MISA software [37]. The search parameters were "1–10 2–6 3–5 4–5 5–5 6–5". The numbers before and after the "-" represent the unit size and minimal numbers of repeats, respectively. Then, we analyzed the tandem repeats with Tandem Repeats Finder (TRF) software [38] with the size of the repeat unit ≥ 7. The parameters were "2 7 7 80 10 50 500 -f -d -m". 2,7,7 means weights for the match, mismatch, and indels, respectively; 80 and 10 mean detection parameters, matching probability Pm = 80 and indel probability Pi = 10; 50 means minimum alignment score; 500 represents maximum period size. Last, the dispersed repeats were analyzed using VMATCH software [39]. The search parameters for dispersed repeats were “-f -p -h 3 -l 30”. The short explanation: -f: compute maximal forward repeats; -p compute maximal palindromes; -h search for repeats up to the given Hamming distance; -l: specify that repeats must have the given length.

Then we used the online tool IRSCOPE (https://irscope.shinyapps.io/irapp/) to compare the genes on the boundaries of the junction sites of the four plastomes from *Codonopsis* and four plastomes from closely related species. IRSCOPE is a generic local genomic visualizer tool designed to reflect the scaled genetic structure of plastome sequences over their respective four regions [40]. The size variation of angiosperm plastomes is primarily due to the expansion and contraction of the IR and SSC boundary regions. This analysis provides insight into the evolutionary differences among species in the *Codonopsis* genus.

### 2.4 Phylogenetic analysis of *Codonopsis* genus

The whole plastome sequences of 18 species from the *Campanulaceae* family were used for phylogenetic analysis, including *Adenophora divaricate* (NC_036221.1) [41], *Adenophora stricta* (NC_036223.1) [41], *Adenophora triphylla* (NC_040857.1) [42], *Campanula punctata* (NC_033337.1) [43], *Campanula takesimana* (KP006497.1) [44], *Campanula zangezura* (NC_057269.1) [45], *Codonopsis lanceolata* (MH018574.1) [27], *Codonopsis minima* (NC_036311.1) [28], *Codonopsis tsinlingensis* (NC_056284.1) [29], *Cyphia angustiloba* (NC_036086.1) [19], *Cyphia banksiana* (NC_036087.1) [19], *Cyphia belfastica* (NC_036088.1) [19], *Leptocodon hirsutus* (NC_049093.1) [46], *Lobelia chinensis* (NC_035370.1) [47], *Lobelia erinus* (NC_036098.1) [19], *Lobelia galpinii* (NC_036071.1) [19], *Carpodetus serratus* (NC_036084.1) [19]. *Carpodetus serratus* was selected as the outgroup. Firstly, we extracted the common genes’ protein-coding sequence (CDS) by phyloSuit [48]. Then the CDS of a total of 68 common genes (*atp*A, *atp*B, *atpE*, *atp*F, *atp*H, *atp*I, *ccs*A, *ce*mA, *mat*K, *ndh*A, *ndh*C, *ndh*D, *ndh*E, *ndh*F, *ndh*G, *ndh*H, *ndh*I, *ndh*J, *pet*A, *pet*B, *pet*D, *pet*G, *pet*L, *pet*N, *psa*B, *psa*C, *psa*I, *psa*J, *psb*A, *psb*B, *psb*C, *psb*D, *psb*E, *psb*F, *psb*H, *psb*I, *psb*J, *psb*K, *psb*L, *psb*M, *psb*N, *psb*T, *psb*Z, *rbc*L, *rpl*2, *rpl*14, *rpl*16, *rpl*20, *rpl*33, *rpl*36, *rpo*A, *rpo*B, *rpo*C1, *rpo*C2, *rps*2, *rps*3, *rps*4, *rps*7, *rps*8, *rps*11, *rps*12, *rps*14, *rps*15, *rps*16, *rps*18, *rps*19, *ycf*1, *ycf*2) were aligned with MAFFT software [49]. We used two methods to construct the phylogenetic tree: the Maximum-likelihood method and the Bayesian Inference method. The Maximum-likelihood tree was built using IQ-TREE [50] and visualized using iTOL (https://itol.embl.de/) [51]. The bootstrap analysis was performed with 1000 replicates using UBBoot [50]. According to the scores of BIC (Bayesian Information Criterion), the best model was TVM+F+R3 for phylogenetic analysis. Bayesian inferences (BI) analysis was performed using MrBayes (v3.2.7) [52], and the best model was chosen using jModelTest (v2.1.0) [53]. The Bayesian inferences (BI) tree was visualized using iTOL.

### 2.5 Comparative analysis of *Codonopsis* plastome structure

We created Dot plots of the plastome sequences of tangshen and *Arabidopsis thaliana* (NC_000932.1) [54], C. *lanceolata* (MH018574.1) [27], *C*. *minima* (NC_036311.1) [28] and *C*. *tsinlingensis* (NC_056284.1) [29], respectively, to identify possible structure variation using the Gepard software [55]. The Mauve [56] software was used to align the sequences to identify intermolecular recombination events of the *Codonopsis* genus, and *C*. *lanceolata* was selected as the reference. The default values were used for all the parameters. To reveal genomic variations, we aligned the four *Codonopsis* plastomes using the mVISTA program with Shuffle-LAGAN mode [57]. Initially, we did this analysis directly with the plastomes we assembled and the other three *Codonopsis* species. We found an inversion in the large single-copy (LSC) region from the Mauve results, so we inverted this region of the tangshen plastome and used it as the reference for further analysis.

Through analyzing the Dotplot and Mauve results, we found two repeat sequences at both ends of the inversion. We first extracted the sequences, including the inversion and its flanking repeat sequences, using extractseq [58]. Then we obtained the reverse complemented sequence of one repeat sequence using revseq [58]. Finally, we aligned these two sequences using MAFFT [49]. GeneDoc [59] was used to visualize the results. To determine whether the repeat sequences are also present in the plastomes of the other three *Codonopsis* species, we used Gepard to get the approximate location of the repeat sequence and then determine its exact locations by BLASTn [60]. Then we extracted the sequences enclosed in these repeat sequences using extractseq [58] and aligned them with MAFFT.

### 2.6 Hypervariable region analysis

To identify the hypervariable regions among the four *Codonopsis* species, we firstly reversed the inversion region in the tangshen plastome. We wrote a custom script to extract the intergenic spacer regions (IGS) from the GenBank files of the four plastomes. We manually removed those IGS loaded across the boundary and in the inverted region and extracted the IGS sequences using extractseq. Then we aligned the extracted sequences using clustalw2 [61] with options “-type = DNA -gapopen = 10 -gapext = 2”. Finally, we calculated the genetic distance of the intergenic regions using the K2p evolution model implemented in the distmat program from the EMBOSS package [58] with the parameters "-nucmethod 2". The threshold value for mapping is 5. It means to visualize the top five results in Fig 7. These five hypervariable regions can be used as molecular markers to distinguish the four *Codonopsis* species.

### 2.7 Identification and validation of molecular markers for species discrimination

*C*. *minima* is a species endemic to Korea [28], and we have not been able to find its cultivation in China. As a result, we could only collect samples from the other three *Codonopsis* species for molecular marker validation. We designed the primers for the five hypervariable regions using the Primer3 program (http://bioinfo.ut.ee/primer3-0.4.0/). The sequences used to design primers are shown in the S11 and S12 Figs in S1 File. PCR amplifications were performed in a final volume of 50 μL with 25 μL 2 Taq PCR Master Mix, 1 μM of each primer, 1 μL template DNA, and 22 μL ddH2O. All amplifications were carried out in a Pro-Flex PCR system (Applied Biosystems, Waltham, MA, USA) under the following conditions: denaturation at 94°C for 2 min, followed by 35 cycles of 94°C for 30 s, at specific annealing temperature (Tm) for 30 s, 72°C for 60 s and 72°C for 2 min as the final extension. PCR amplicons were visualized on 1.2% agarose gels and then subjected to Sanger sequencing on an ABI 3730XL instrument (Applied Biosystems, USA) using the same primers used for PCR amplification.

## 3. Results

### 3.1 General features of the plastome

**Fig 1:**
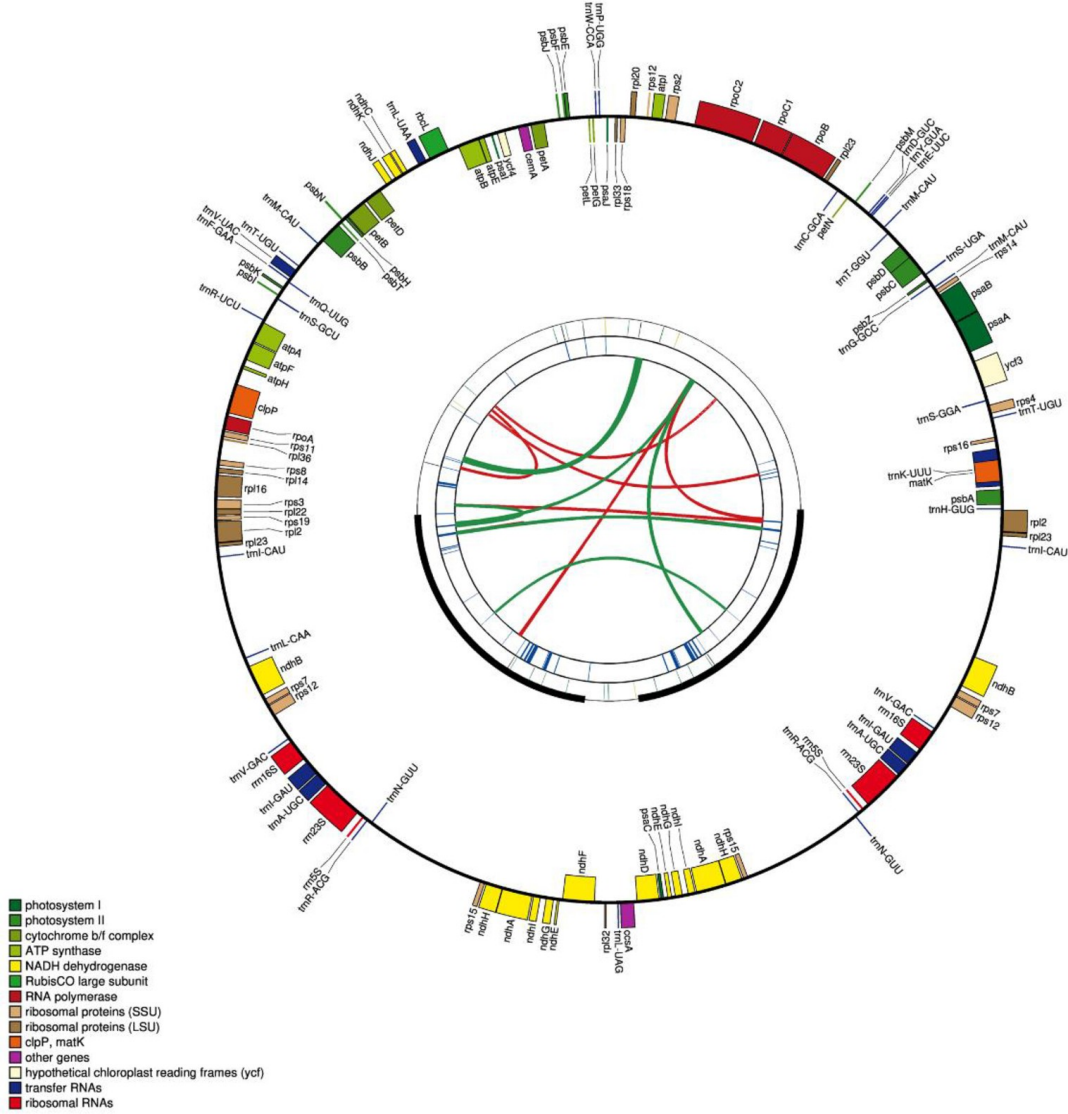
The schematic representation of the plastome of *C*. *pilosula* subsp. *tangshen* created by CPGAVAS2. The map contains four rings. From the center to outward, the first circle represents the forward and reverse repeats indicated by red and green arcs, respectively. The second circle represents the tandem repeats marked with short bars. And the third circle shows the microsatellite sequences identified using MISA. The fourth circle shows the gene structure on the plastome. The genes were colored based on their functional categories, which are shown in the left corner.

The tangshen plastome was a circular sequence, showing a typical quadripartite structure. It was 170,672 bp long in length and consisted of an 86,108 bp large single-copy (LSC) region, a 7,654 bp small single-copy (SSC) region, and a pair of 38,455 bp long identical inverted repeats (IRs) (Fig 1). There are 108 genes in the tangshen plastome, including 76 protein-coding genes, 28 tRNA genes, and four rRNA genes (Table 1). Among these genes, there are 14 genes (*trn*K-UUU, *trn*L-UAA, *trn*V-UAC, *atp*F, *pet*B, *pet*D, *rpl*2, *ycf*2, *rpl*16, *rps*19, *ndh*B, *trn*I-GAU, *trn*A-UGC, *ndh*A) containing one intron, three genes (*ycf*3, *clp*P, *ycf*1) containing two introns (S2 Table in S1 File). There are ten cis-splicing genes (*trn*K-UUU, *ycf*3, *rpo*C1, *atp*F, *trn*L-UAA, *pet*D, *rpl*16, *rpl*2, *trn*E-UUC, *trn*E-UUC) in the tangshen plastome (S1 Fig in S1 File), including six protein-coding genes and three tRNA genes. Except that the *ycf*3 gene has two introns and three exons, other genes have one intron and two exons. Two *trn*E-UUC genes are cis-spliced. Only the *rps*12 gene is trans-spliced. There are two copies of the *rps*12 gene. Each copy has three exons (S2 Fig in S1 File).

**Table 1:** Gene compositions of the *Codonopsis pilosula* subsp. *tangshen* plastome.

Category of genes	Group of genes	Name of genes
	rRNA	*rrn*4.5S (×2), *rrn*5S (×2), *rrn*16S (×2), *rrn*23S (×2)
	tRNA	*trn*A-UGC (×2), *trn*C-GCA, *trn*D-GUC, *trn*E-UUC, *trn*F-GAA, *trn*G-GCC, *trn*H-GUG, *trn*I-CAU (×2), *trn*I-GAU (×2), *trn*K-UUU, *trn*L-CAA, *trn*L-UAA, *trn*L-UAG, *trn*M-CAU(x3), *trn*N-GUU(x2), *trn*P-UGG, *trn*Q-UUG, *trn*R-UCU, *trn*R-ACG(x2), *trn*S-GCU, *trn*S-GGA, *trn*S-UGA, *trn*T-GGU, *trn*T-UGU (×2), *trn*V-GAC(x2), *trn*V-UAC, *trn*W-CCA *trn*Y-GUA
photosynthesis	Subunits of ATP synthase Subunits of photosystem II	*atp*A, *atp*B, *atp*E, *atp*F, *atp*H, *atp*I
		*psb*A, *psb*B, *psb*C, *psb*D, *psb*E, *psb*F, *psb*I, *psb*J, *psb*K, *psb*L, *psb*M, *psb*N, *psb*T, *psb*Z, *ycf*3
	Subunits of cytochrome b/f complex	*pet*A, *pet*B, *pet*D, *pet*G, *pet*L, *pet*N
	Subunits of photosystem I	*psa*A, *psa*B, *psa*C, *psa*I, *psa*J
	Subunit of rubisco	*rbc*L
	Subunits of NADH-dehydrogenase	*ndh*A(×2), *ndh*B(×2), *ndh*C, *ndh*D, *ndh*E(×2), *ndh*F, *ndh*G(×2), *ndh*H(×2), *ndh*I(×2), *ndh*J, *ndh*K
Self-replication Other genes	Large subunit of ribosome	*rpl*14, *rpl*16, *rpl*2(×2), *rpl*20, *rpl*22, *rpl*23(×3), *rpl*32, *rpl*33, *rpl*36
	DNA dependent RNA polymerase	*rpo*A, *rpo*B, *rpo*C1, *rpo*C2
	Small subunit of ribosome	*rps*11, *rps*12(×2), *rps*14, *rps*15(×2), *rps*16, *rps*18, *rps*19, *rps*2, *rps*3, *rps*4, *rps*7(×2), *rps*8
	c-type cytochrome synthesis gene	*ccs*A
	Envelop membrane protein	*cem*A
	Protease	*clp*P
	Maturase	*mat*K
Unknown	Conserves open reading frames	*ycf*1(×2), *ycf*2(×2), *ycf*4, *ycf*15(×2)

The length of the protein-coding sequence (CDS) in the tangshen plastome is 89,442 bp, representing 52.41% of the entire length. In contrast, the size of rRNA is 9,063 bp, and tRNA is 2,769 bp, representing 5.31% and 1.62% of the total length of the tangshen plastome sequence, respectively. The GC content analysis showed that the overall GC content is 38.15%, whereas GC contents for the CDS, rRNA, and tRNA genes are 38.40%, 54.76%, and 52.87%, respectively. In contrast, the GC contents for the LSC, SSC, and IRs are 36.75%, 32.07%, and 40.31%, respectively. Moreover, a total of 50,591 codons were identified in the tangshen plastome. In total, 64 codons encode 20 amino acids and three termination codons. Among these codons, 5,325 codons encode leucine, and 656 codes encode cysteine, representing the most and least abundant coded amino acids in the tangshen plastome (S3 Table in S1 File).

**Fig 2:**
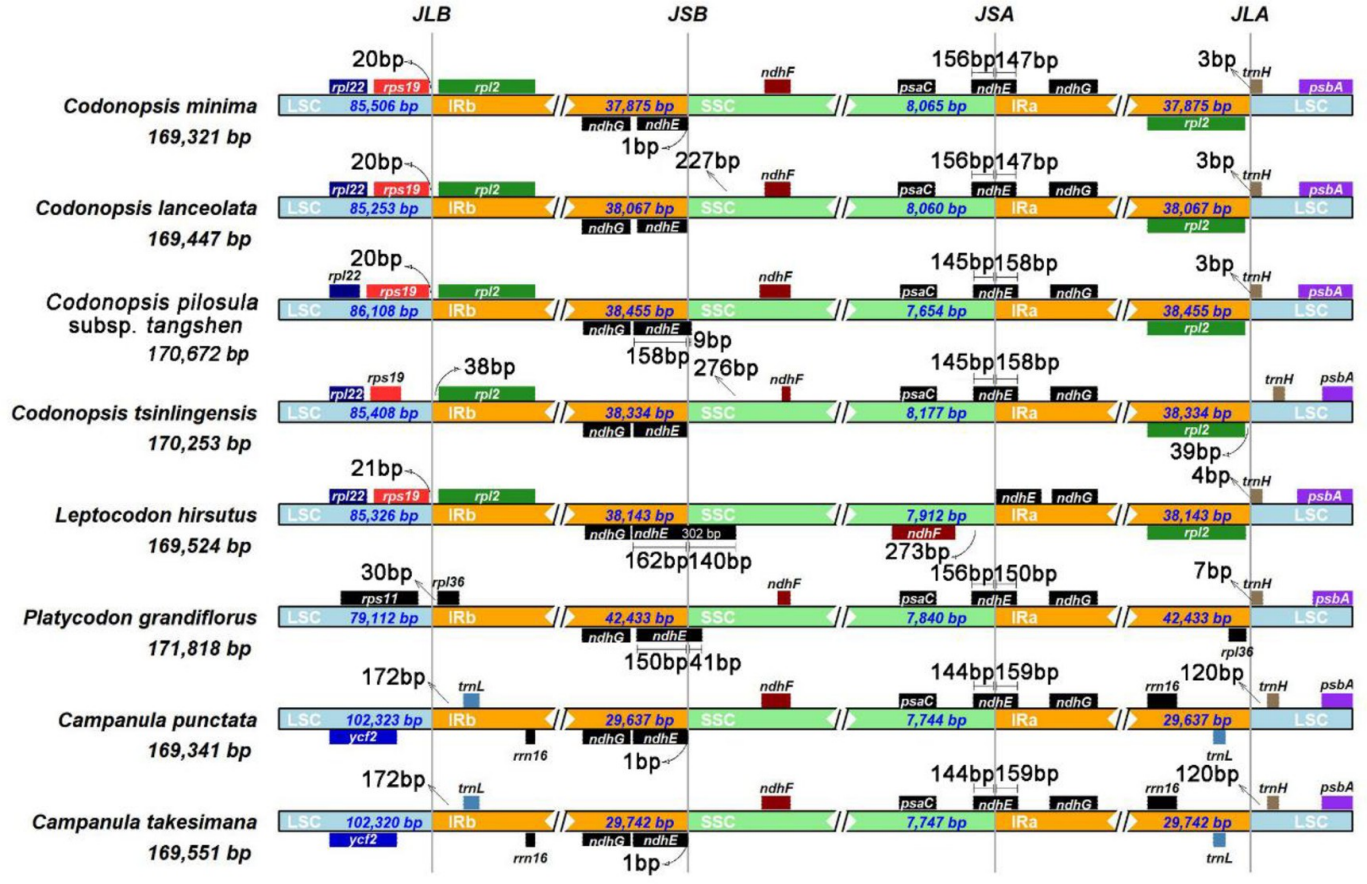
Comparison of LSC, IR, and SSC border among the complete plastomes of 4 *Codonopsis* species, 2 *Campanula* species, *Leptocodon hirsutus*, and *Platycodon grandiflorus*. The JLB, JSB, JSA, and JLA represent junction sites of LSC/IRb, IRb/SSC, SSC/IRa, IRa/LSC, respectively. Different colors represent different regions. The blue represents LSC, the orange represents IRs, and the green represents SSC. The number on the arrow indicates the distance between the gene and the boundary. The genes shown in Fig are the genes closest to the boundary.

The IRs are the most conserved regions of the plastome, and their boundary often undergoes contraction and expansion. As shown in Fig 2, the length of LSC, SSC, and IRs differ among the four *Codonopsis* plastomes. The sizes of IRs in the *Codonopsis* plastomes range from 37,875 to 38,455 bp. There are eleven genes (*rpl*22, *rps*19, *rpl*2, *ndh*G, *ndh*F, *psa*C, *ndh*E, *ndh*G, *rpl*2, *trn*H, *psb*A) at the four junctions: JLB (LSC/IRb), JSB (IRb/SSC), JSA (SSC/IRa) and JLA (IRa/LSC). The JLB junction of the four *Codonopsis* plastomes was located between the genes *rps*19 and *rpl*2. However, the gene *rps*19 in *C*. *tsinlingensis* was further away from the JLB junction compared with those of the other three *Codonopsis* species.

The *ndh*E was found in both JSA and JSB junctions. In both *C*. *lanceolata* and *C*. *minima*, 156 bp of the *ndh*E gene locates in the SSC, and 147 bp locates in the IRa. In both tangshen and *C*. *tsinlingensis*, 145 bp of *ndh*E genes were located in the SSC, and 158 bp of the genes were located in the IRa. The JLA junctions were located to the right of the *rpl*2 and the left of the *trn*H in the four *Codonopsis* plastomes. The *trn*H in *C*. *tsinlingensis* is further away from the junction.

### 3.2 Repeat analysis

We analyzed three kinds of repeat sequences (microsatellite repeats, tandem repeats, and dispersed repeats) in the tangshen plastome. We identified 30 microsatellite repeats (S4 Table in S1 File), including 19 mononucleotides (A/T), eight dinucleotides (AT/AT), and three trinucleotide repeats (2 AGG/CCT, and 1 AAG/CTT). Among them, 14 microsatellite repeats in the protein-coding regions (*rpo*C1, *rpo*C2, *cem*A, *clp*P, *ycf*1, *ndh*A). There are 40 tandem repeats in the tangshen plastome (S5 Table in S1 File), meeting the two conditions that the length of the repeat unit is more than 30 bp and the similarity among the repeat unit sequences is more than 90%. The lengths of repeat units range from 30 bp to 315 bp. For the dispersed repeats, 49 were identified, containing 27 palindromic repeats and 22 direct repeats (S6 Table in S1 File). The longest and shortest dispersed repeat units are 540 bp and 148 bp, respectively.

### 3.3 Phylogenetic analysis

**Fig 3:**
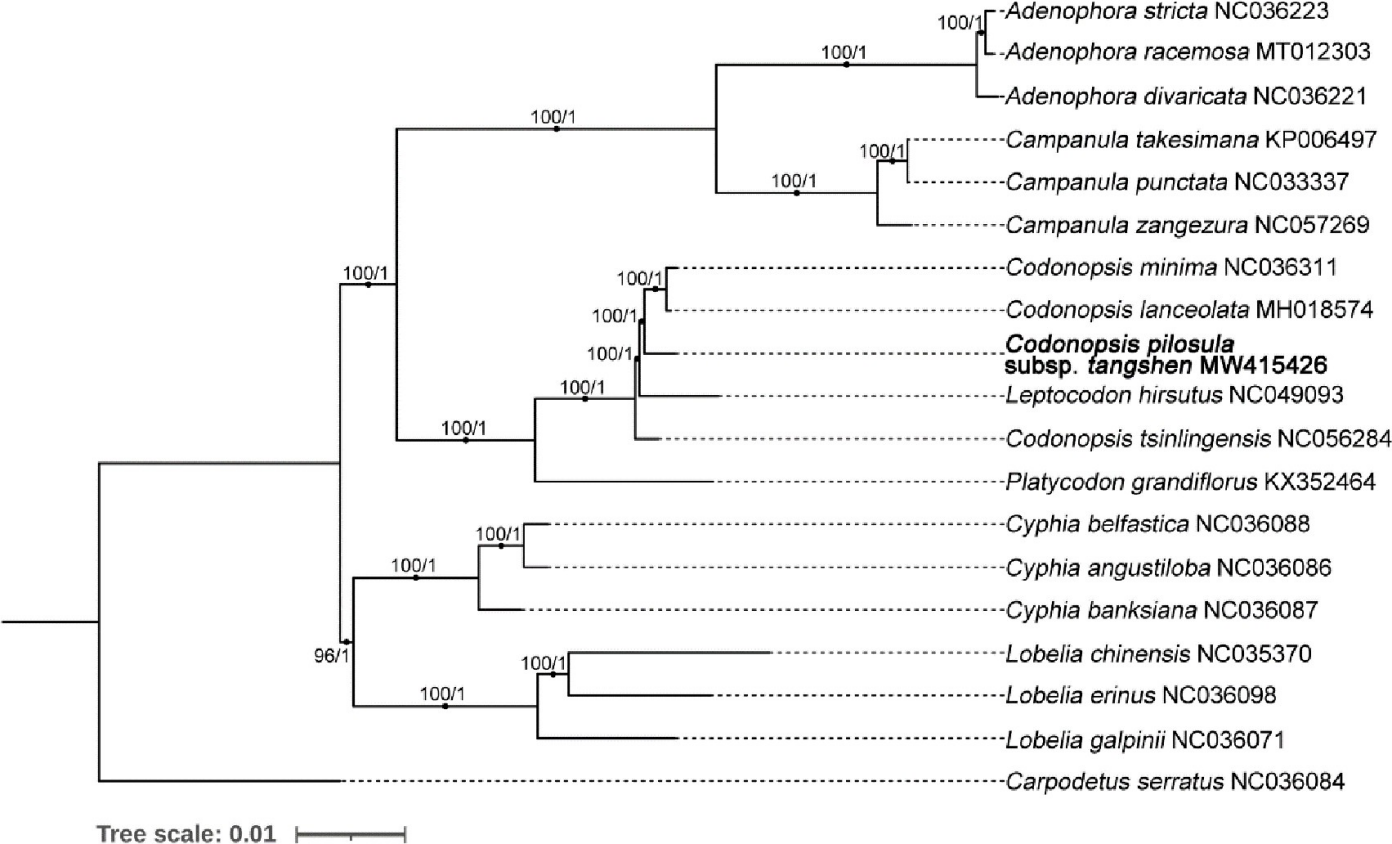
The phylogenetic tree of species from *Codonopsis* and other genus constructed based on the nucleotide sequences of 68 conserved plastid protein-coding genes using the maximum likelihood (ML) method and Bayesian Inference (BI) method. The number next to each node represents the corresponding bootstrap value and the BI posterior probabilities, respectively. The GenBank accession number are shown after the Latin name of the species. The sequence obtained from this study was highlighted in Bold. The length of the branch corresponds to the frequency of base substitutions.

To construct the phylogenetic tree, we selected 19 plastome sequences from the genera of *Adenophora*, *Campanula*, *Codonopsis*, *Leptocodon*, *and Platycodon*, *Cyphia*, *Lobelia*, and *Carpodetus* (Fig 3). The *Carpodetus serratus* was selected as the outgroup taxa. The phylogenetic trees were constructed using the maximum likelihood (ML) method and Bayesian Inference (BI) method. The results of both methods had the same topological structure. The 15 species were divided into two main clades. In particular, the *Cyphia* and *Lobelia* species formed one clade, and the other 12 species formed another clade. Then *Adenophora* and *Campanula* species formed a clade, four *Codonopsis* species, one *Platycodon* species, and one *Leptocodon* species formed another clade. Interestingly, *Leptocodon hirsutus* is more closely related to the other three *Codonopsis* species than *Codonopsis tsinlingensis*. Therefore, the most closely related species is *Leptocodon hirsutus* in this phylogenetic tree. The bootstrap support scores for all branches were more than 96 in the tree built by the maximum likelihood (ML) method. The Bayesian inference (BI) posterior probabilities for all branches are 1, showing the high reliability of this tree.

### 3.4 Structure variation of tangshen plastome

**Fig 4:**
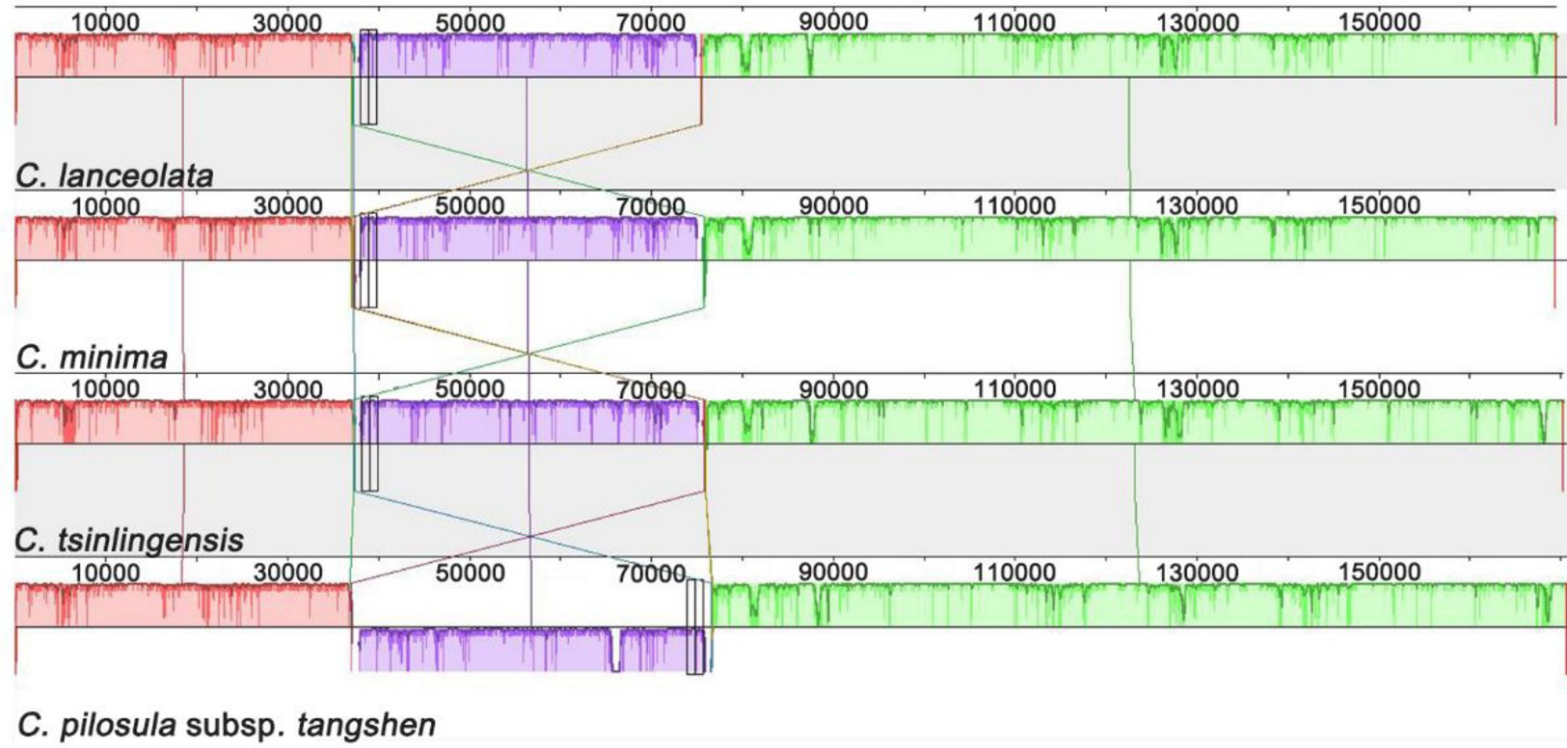
Structure variation in *C*. *pilosula* subsp. *tangshen*. A comparison of tangshen plastome from this study and *C*. *lanceolata*, *C*. *minima*, and *C*. *tsinglingensis* from NCBI revealed similarities and differences in syntenic blocks. The box in purple indicates the correspondence of the region in different species. The *C*. *pilosula* subsp. *tangshen* was used as the reference. An inversion was found in *C*. *pilosula* subsp. *tangshen* was shown in purple.

The tangshen plastome was compared with those of *A*. *thaliana*, *C*. *lanceolata*, *C*. *minima*, *and C*. *tsinlingensis* for structural variations. The Dotplot results showed a high degree of collinearity between the *Codonopsis* and *A*. *thaliana* plastomes (S3-S7 Figs in S1 File). Mauve alignment of plastomes indicated that the tangshen plastome has an unusually large inversion in the LSC region than the other three *Codonopsis* plastomes (Fig 4). Then we compared the genes in the inversion region of the four *Codonopsis* species. We found the genes from *atp*H to *rps*2 are inverted in the tangshen plastome. The exact positions of this inversion are from 37,849 to 75,896.

**Fig 5:**
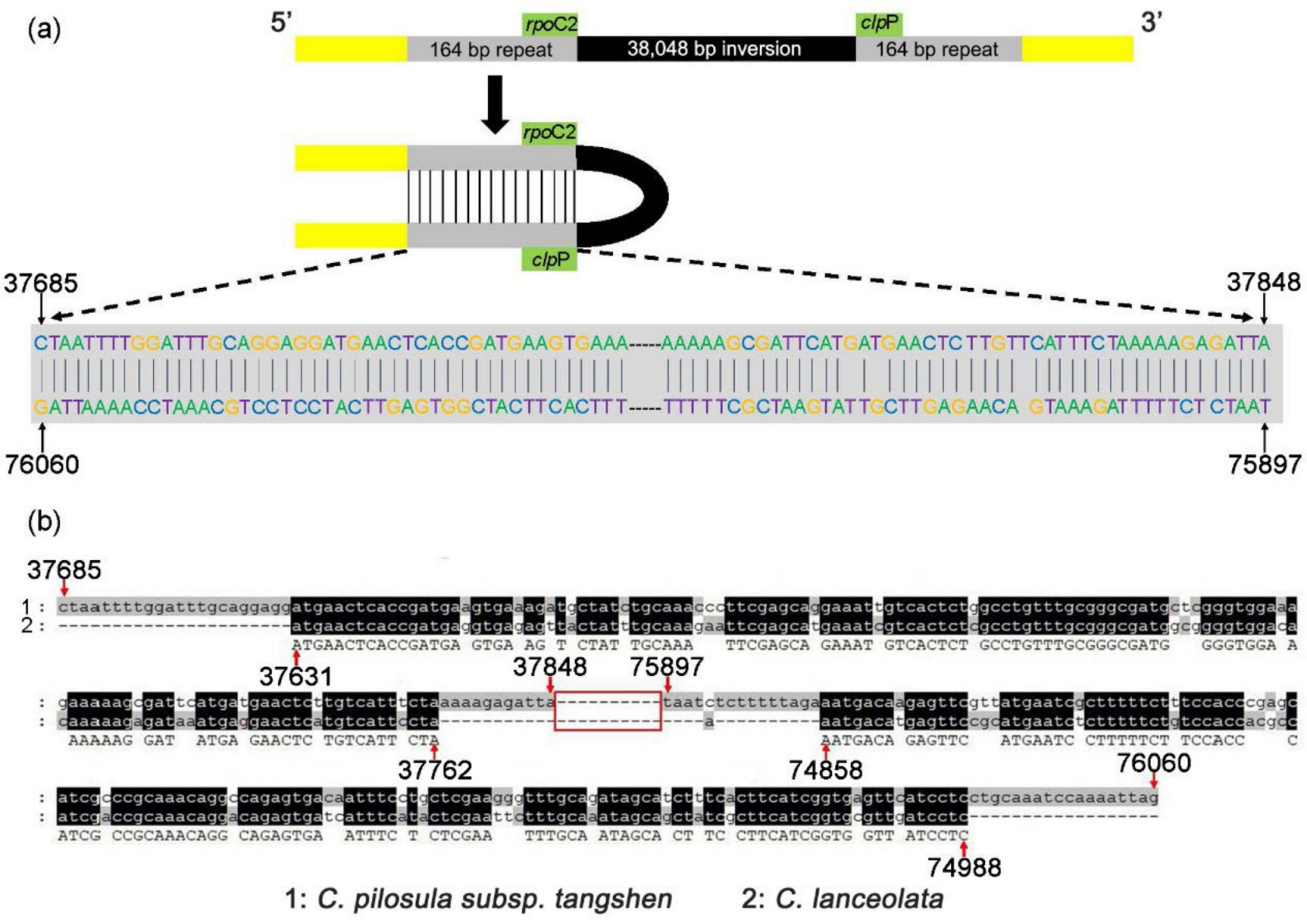
Alignment of two repeat sequences at both ends of the inversion in *C*. *pilosula* subsp. *tangshen*. **(a)** The schematic representation of the inversion region and the two repeat sequences. The black shaded area represents the inversion region. Grey areas links repeat sequences at both ends. The yellow areas represent the sequences at both ends of the repeat sequences. *rpo*C2 and *clp*P represent the genes closest to the inversion region. The direction was from 5’ to 3’. The number on the arrows represents the starting and ending positions of the two repeat sequences, respectively. The dash in the middle of the sequences represents the omitted sequence. **(b)** Alignment of the repeat sequences in *C*. *pilosula* subsp. *tangshen* and *C*. *lanceolata*. The black shading represents the two repeat unit sequences. The two repeat units are palindromic. The red box in the middle represents the omitted sequences between the two repeat units. The numbers pointed by the red arrows represent the start and end positions of the two repeat units in *C*. *pilosula* subsp. *tangshen* and *C*. *lanceolata*. The repeat sequence in *C*. *pilosula* subsp. *tangshen* is longer than that in *C*. *lanceolata*. The code for the species was listed below this Fig.

Compared with the dispersed repeats shown in S7 Table in S1 File, we found one pair of dispersed repeat sequences flanking the inversion with a length of 164 bp. The alignment of these two repeat sequences is shown in Fig 5A. These two repeat sequences are reverse complementary, forming a palindromic repeat. The repeat unit on the 5’ end is from 37,685 to 37,848. And the repeat unit on the 3’ end is from 75,897 to 76,060. We further checked whether this repeat sequence was also present in the other three *Codonopsis* species. We found this repeat sequence also exists in the *C*. *lanceolata* plastome, but the length of 132 bp is shorter (Fig 5B). We did not find any repeat sequences in the plastomes of *C*. *minima* and *C*. *tsinlingensis* at similar positions. The repeat sequence in the tangshen plastome is likely to be involved in the genesis of the large inversion. However, the repeat sequence did not generate any inversion in the *C*. *lanceolata* plastome.

**Fig 6:**
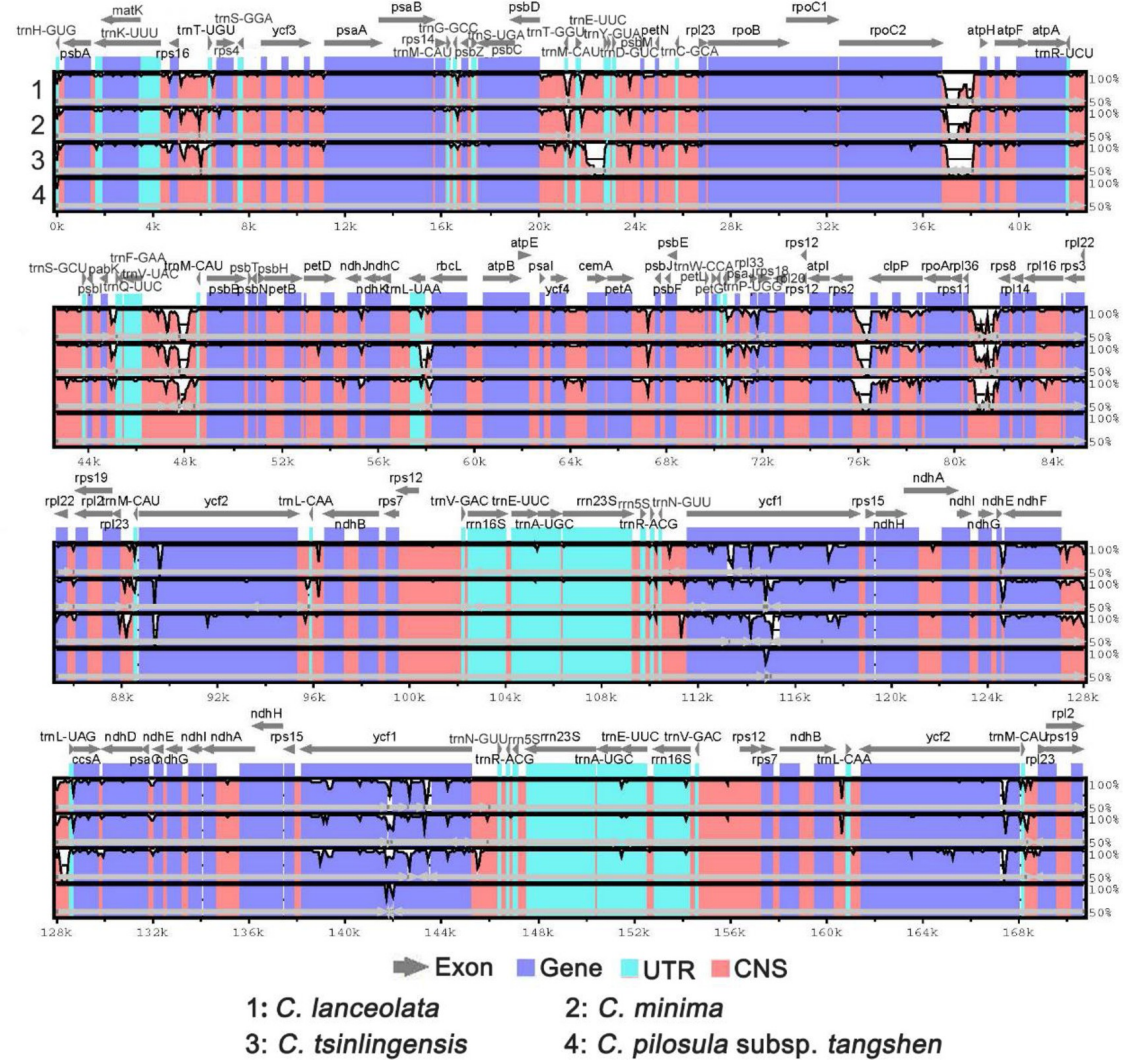
Comparison of the four *Codonopsis* plastomes by mVISTA. The vertical scale on the right indicates the percentage of identity, ranging from 50 to 100%. The horizontal axis shows the coordinates within the plastome. Gray arrows indicate the genes above the alignments. Different colors represent different regions. The dark blue, light blue, and pink represent exon, tRNAs, or rRNAs and conserved non-coding sequences. The reference is the *C*. *pilosula* subsp. *tangshen*, with its inversion region inverted for comparison. The number code for the species was shown below the picture.

We manually inverted the inversion to compare the sequences and used the inverted tangshen plastome as the reference. (Fig 6). The IRs of the four plastomes showed relatively lower sequence similarity than the LSC and SSC regions. Among the four different functional regions: exon, intron, gene, and conserved non-coding sequences (CNS), the CNS regions showed the highest variations. The exon regions of the four plastomes generally exhibited relatively higher conservation than the CNS and intron regions. Still, the two copies of *the ycf*1 gene showed a higher degree of variations among the four plastomes. In the IRs regions, the *rrn*23 gene with two copies exhibited the highest similarity among the four plastomes. Overall, the tangshen sequences exhibited relatively higher levels of sequence divergence among the *Codonopsis* species.

### 3.5 Analysis of hypervariable regions

**Fig 7:**
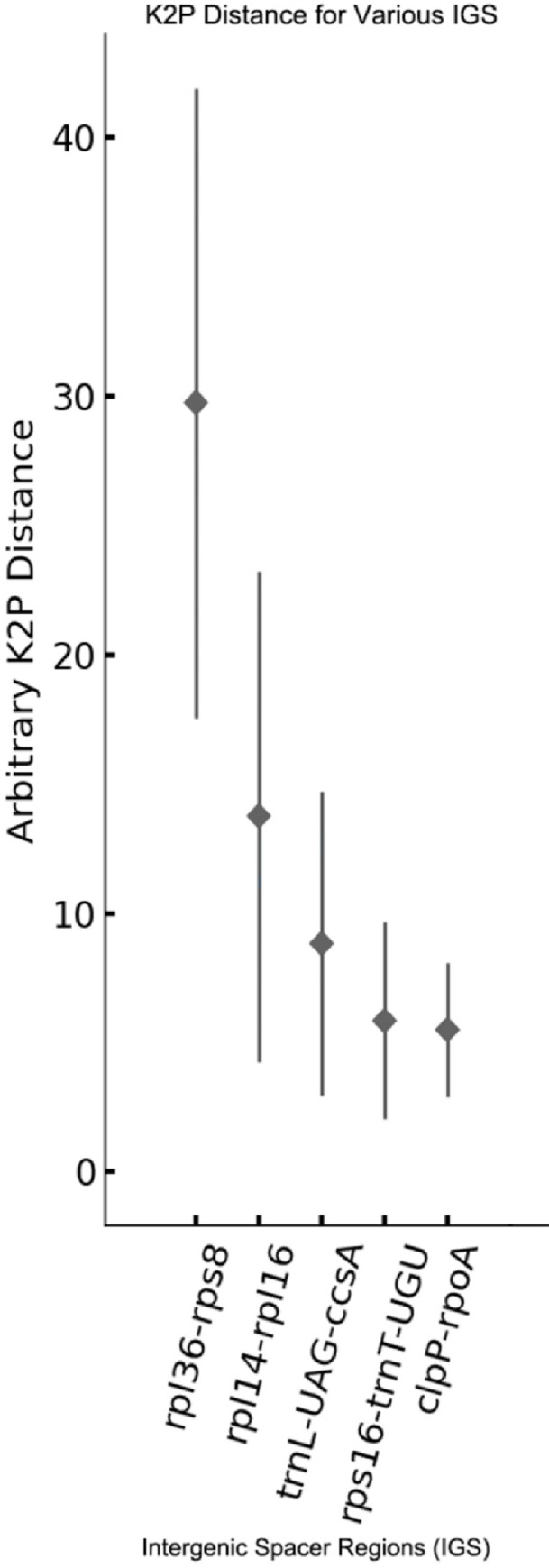
The hypervariable regions between the *Codonopsis* genus. The horizontal direction represents the intergenic spacer regions that are highly variable among the four *Codonopsis* species. The vertical direction is the arbitrary K2P distance of these regions. The square in the middle of each line represents the main distance of each intergenic spacer region.

Five hypervariable regions having the highest variations were shown in Fig 7. The five regions: *rpl*36-*rps*8, *rpl*14-*rpl*16, *trn*L-UAG-*ccs*A, *rps*16-*trn*T-UGU, and *clp*P*-rpo*A had the K2p values 29.74, 13.75, 8.83, 5.87, and 5.49, respectively. And these five hypervariable regions can be used as the potential molecular markers to distinguish these four *Codonopsis* species.

### 3.6 Development of molecular markers from the plastomes

To discriminate the available three *Codonopsis* species, we named the DNA marker from the five hypervariable regions *rpl*36-*rps*8, *rpl*14-*rpl*16, *trn*L-UAG-*ccs*A, *rps*16-*trn*T-UGU, and *clp*P*-rpo*A as *Codonopsis* marker 1–5, and Com1, Com2, Com3, Com4, and Com5, in short, respectively. The PCR primers used to amplify these five markers are shown in the S7 Table in S1 File. The Com2 and Com5 failed for PCR amplification. The Com3 sequences amplified from the three species had only one SNP and could not distinguish the three species. The three markers will not be discussed any further.

**Fig 8:**
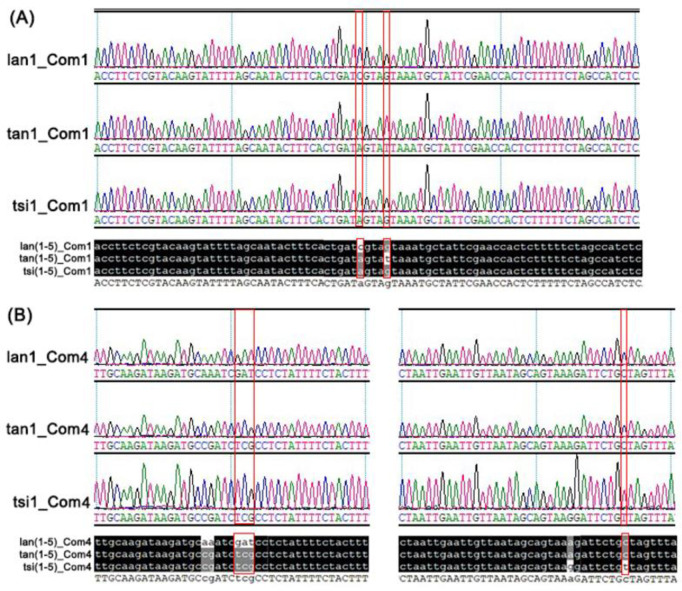
The alignment of the sequencing chromatogram of the PCR products was amplified using the primers of Com1 and Com4. The ID of each sequence is shown on the left side of each panel. The composition of the ID in turn includes the abbreviation of the species name, plant individual id, and primer name. The figure of alignment represents the individuals 1–5 of three species. The sequencing chromatogram of the PCR products amplified takes individual 1 of three species as an example. The red squares represent the SNP and Indel regions, which can distinguish the three species. The nucleotides identical across all plastomes are shaded in black, whereas those conserved in 60% of the sequences are shaded in gray. lan: *Codonopsis lanceolata*; tan: *Codonopsis pilosula* subsp. *tangshen*; tsi: *Codonopsis tsinlingensis*. Arabic numerals represent the individual 1.

The product sizes of PCR amplification of Com1, Com3, and Com4 markers from the three *Codonopsis* species were similar to expected (S8 Fig in S1 File). The DNA fragments were extracted from each band and then subjected to Sanger sequencing. The sequencing results for the PCR products of Com1 and Com4 were identical to the expected sequences (S9 and S10 Figs in S1 File). The marker Com1, derived from the *rpl*36-*rps*8 region, has seven specific SNP loci. The first SNP loci shown in red squares can be used to differentiate two of the three *Codonopsis* species, except *C*. *lanceolata*. The second SNP loci, which are shown in red squares, can be used to differentiate tangshen and *C*. *tsinlingensis* (Fig 8A, S9 Fig in S1 File). The marker Com4 is derived from the *rps*16-*trn*T-UGU IGS region. It has five SNP loci and two Indel loci. Combined with the SNP and Indel loci shown in red squares can be used to distinguish the three *Codonopsis* species (Fig 8B, S10 Fig in S1 File). We also have tested the new primers on all four available *Codonopsis* plastomes obtained from NCBI and this study. These markers can discriminate all four species based on the SNP and Indel loci from Com1 and Com4 (S11 and S12 Figs in S1 File).

## 4. Discussion

In this study, we first reported the complete plastome of *Codonopsis pilosula* subsp. *tangshen* and then made a systematic comparative analysis of the *Codonopsis* plastomes. Notably, we have (1) sequenced and assembled the tangshen plastome; (2) compared the four *Codonopsis* plastomes and found an unusually large inversion having two repeat sequences at both ends of the inversion in the tangshen plastome; (3) identified the top five hypervariable regions for the development of potential molecular markers and validated two of them successfully; (4) carried out the phylogenetic analysis of *Codonopsis* and its relative genus.

Accurate identification of herbal medicines helps ensure the safe and effective use of herbal medicines. DNA-based markers are suitable for the discrimination of different medicinal plants [62]. Three kinds of DNA barcode markers are of importance: conventional, super, and specific markers. The conventional markers are universal and are applied to all medicinal plants, including ITS2, *psb*A-*trn*H, *rbc*L, and *mat*K. This group of markers can be used as the first line of tools to discriminate samples.

The success of species discrimination of these universal markers is mostly for distantly related species. It generally lacks the discrimination powers for closely related species. In these cases, universal markers with higher-resolution or taxon-specific markers are needed. The complete plastomes have been acclaimed as a super barcode to distinguish related species, especially for taxonomically difficult taxa [26]. The super barcodes demonstrated high discriminative power and sufficient reliability in the previous study.

However, its use may be limited due to the insufficient amount of available DNA to assemble the complete genome, expensive sequencing costs to generate enough raw data to assemble the complete genome, and the complexity of data analysis. Therefore, searching for specific barcodes from hypervariable regions is important as a trade-off between universal and super DNA barcodes [21].

Several successful examples have been reported to develop taxon-specific markers [63]. In the present study, the universal makers have not been successful in discriminating the medicinal *Codonopsis* species. As a result, we sequence the plastome sequences and developed taxa-specific makers for the discrimination.

In addition to serving as the source of specific DNA barcodes, the plastomes can also be used to understand the phylogenetic relationship of closely related species. The phylogenetic relationship identified in this study is similar to those described previously. Classifications analyses among *Codonopsis* were previously reported based on four chloroplast gene regions: *rbc*L, *mat*K, *trn*H-*psb*A, and the nuclear internal transcribed spacer (nrITS) [12].

Another application of the complete plastome is the identification of unusual structures that reflect the evolutionary history of the study subject. This study performed an in-depth comparative analysis of the four *Codonopsis* plastomes. We found a large inversion in the LSC region in the tangshen plastome. Interestingly, we found a pair of palindromic repeat sequences flanking this inversion. A similar repeat sequence was also present in the plastome of *C*. *lanceolata*, but the repeat length was 32 bp shorter. Similar repeat sequences were not found in the other two *Codonopsis* species’ plastomes at similar locations. And no inversions were found in the other three *Codonopsis* plastomes.

We proposed two models for the genesis of the inversion in tangshen plastome. The first evolutionary model is that the repeat sequence found in the four *Codonopsis* plastomes was acquired before the speciation of the *Codonopsis* genus. During the differentiation of *Codonopsis* species, repeat sequences were further differentiated. The tangshen repeat sequence remains active, leading to rearrangement. However, the repeat sequences experience partial or complete deletion in the other three *Codonopsis* species, preventing the formation of the palindromic structure and the generation of the inversion. The second model is that the repeat sequence was acquired after the *Codonopsis* speciation independently. From a parsimonious point of view, the second model is less likely to be true. Another interesting question is whether or not the inversion occurred a long time ago. With the availability of more plastomes, we can calculate the percentage of plastomes having and not having the inversions, which might help answer this question.

Inversions mediated by palindromic repeat are not rare in the plastome [64]. The Campanulaceae species mainly consist of perennial herbs and have the most plastome structural variants based on previous studies on enzymatic loci, gene localization, and genome sequencing [2–5]. For example, previous studies have found numerous structural changes in the *Adenophora* and *Trachelium* species [4, 6]. Therefore, studying the plastome structure is essential to understanding the phylogenetic relationships and evolutionary history among Campanulaceae species [7].

Previous studies have reported that genomic rearrangements occur due to incorrect recombination of repeat sequences and mispairing of sliding strands [65, 66]. So the repeat sequences play a crucial role in plastome rearrangement [67]. For example, the *psb*A*-trn*H intergenic region is well known to have a small inversion [68]. This region is frequently used for DNA barcode analysis to distinguish different species [69]. It is generally thought that the palindromic repeat can form a stem-loop structure. Some exonucleases will cut the single-stranded DNA. When the DNA repair system is coming to repair the incised single-stranded DNA, it might connect the wrong DNA strand, forming an inversion (Fig 5A).

One limitation of the current study is the small number of *Codonsopsis* plastomes analyzed. The plastomes of only four of 42 *Codonopsis* species have been reported. Consequently, the results obtained from this study will only apply to the four *Codonopsis* species. Unfortunately, we could collect samples from three of the four species. The *C*. *minima* was an endemic species to Korea [28] and could not be found in China. Therefore, the molecular markers validated in this study might only help discriminate between these three species. More plastomes of *Codonopsis* species are needed in the future to elucidate the taxonomic classification and evolutionary history of the *Codonopsis* species. It should be pointed out that the three species we have analyzed have medicinal values and are most likely to be used indiscriminately.

## 5. Conclusions

We sequenced and analyzed the plastome of tangshen. Based on the four *Codonopsis* plastomes, we identified and validated two molecular markers using PCR amplification and Sanger sequencing experiments. Comparative analysis showed that the four *Conodopsis* plastomes have a lower level of genetic diversity. The tangshen plastome has a unique inversion, likely to be formed by repeat-mediated rearrangement.

## Supporting information

S1 File (DOCX)
