# Supplementary material for: Characterization of Codonopsis pilosula subsp. tangshen plastome and comparative analysis of Codonopsis species
Source: PLoS One. 2022 Aug 1;17(8):e0271813. doi: 10.1371/journal.pone.0271813 (PMC9342729; doi:10.1371/journal.pone.0271813)
Supplement: S1 File — (DOCX) [file pone.0271813.s001.docx]

# Title:

Characterization of *Codonopsis pilosula* subsp. *tangshen* plastome and comparative analysis of *Codonopsis* species

# Author list

Jingwen Yue^1¶^, Yang Ni^1¶^, Mei Jiang^2^, Haimei Chen^2^, Pinghua Chen^1^*, Chang Liu^2^*

# Affiliations

^1^Key Laboratory of Ministry of Education for Genetics, Breeding and Multiple Utilization of Crops, National Engineering Research Center of Sugarcane, College of Agriculture, Fujian Agriculture and Forestry University, Fuzhou, Fujian Province, Fuzhou 350002, P. R. China;

^2^Key Laboratory of Bioactive Substances and Resource Utilization of Chinese Herbal Medicine from Ministry of Education, Engineering Research Center of Chinese Medicine Resources from Ministry of Education, Institute of Medicinal Plant Development, Chinese Academy of Medical Sciences, Peking Union Medical College, Beijing 100193, P. R. China.

*****Correspondence: Pinghua Chen: [phcemail@126.com](mailto:phcemail@126.com) (PHC); Tel: +86-0591-83789177, Fax: +86-0591-83768242; Chang Liu: [cliu6688@yahoo.com](mailto:cliu6688@yahoo.com) (CL); Tel: +86-10-57833111, Fax: +86-10- 62899715;

^¶^These authors contributed equally to this work.

E-mails:

Jingwen Yue: [yjwbisheng@163.com](file:///C:\Users\Administrator\AppData\Roaming\Microsoft\Word\yjwbisheng@163.com)

Yang Ni: [ny_work@126.com](mailto:ny_work@126.com)

Mei Jiang: [mjiang0502@163.com](file:///C:\Users\Administrator\Desktop\文章\党参\A川党参文章修改版本\yjw-tangshen-20210329\mjiang0502@163.com)

Haimei Chen: [hmchen@implad.ac.cn](mailto:hmchen@implad.ac.cn)

Pinghua Chen: [phcemail@126.com](mailto:phcemail@126.com)

Chang Liu: [cliu6688@yahoo.com](mailto:cliu6688@yahoo.com)

# S1 Table. The list of the samples used in the sequencing and species discrimination analyses of the *Codonopsis*.

| Purpose | Species | Sample No. | Location collected | GPS |
| --- | --- | --- | --- | --- |
| Sequencing | tangshen | Implad201808044 | Enshi, Hubei | 109°76’ E, 30°18’ N |
| Molecular marker validation | *C. lanceolata* | lan1 | Huanggang, Hubei | 114°87’ E, 30°44’ N |
|  |  | lan2 | Huanggang, Hubei | 114°87’ E, 30°44’ N |
|  |  | lan3 | Huanggang, Hubei | 114°87’ E, 30°44’ N |
|  |  | lan4 | Huanggang, Hubei | 114°87’ E, 30°44’ N |
|  |  | lan5 | Huanggang, Hubei | 114°87’ E, 30°44’ N |
|  | tangshen | tan1 | Enshi, Hubei | 109°76’ E, 30°18’ N |
|  |  | tan2 | Enshi, Hubei | 109°76’ E, 30°18’ N |
|  |  | tan3 | Enshi, Hubei | 109°76’ E, 30°18’ N |
|  |  | tan4 | Enshi, Hubei | 109°76’ E, 30°18’ N |
|  |  | tan5 | Enshi, Hubei | 109°76’ E, 30°18’ N |
|  | *C. tsinlingensis* | tsi1 | Qinling, Shanxi | 107°94’ E, 33°98’ N |
|  |  | tsi2 | Qinling, Shanxi | 107°94’ E, 33°98’ N |
|  |  | tsi3 | Qinling, Shanxi | 107°94’ E, 33°98’ N |
|  |  | tsi4 | Qinling, Shanxi | 107°94’ E, 33°98’ N |
|  |  | tsi5 | Qinling, Shanxi | 107°94’ E, 33°98’ N |

# S2 Table. The lengths of introns and exons for the splitting genes in the tangshen plastome.

| Gene | Strand | Start | End | Exon I | Intron I | Exon II | Intron II | Exon III |
| --- | --- | --- | --- | --- | --- | --- | --- | --- |
| *trn*K-UUU | - | 1620 | 4245 | 37 | 2554 | 35 |  |  |
| *ycf*3 | + | 8526 | 10473 | 129 | 695 | 230 | 743 | 151 |
| *trn*L-UAA | + | 55766 | 56357 | 35 | 507 | 50 |  |  |
| *pet*D | - | 59572 | 60777 | 8 | 720 | 478 |  |  |
| *pet*B | - | 60971 | 62435 | 6 | 817 | 642 |  |  |
| *trn*V-UAC | + | 67592 | 68233 | 38 | 569 | 35 |  |  |
| *atp*F | - | 73416 | 74707 | 145 | 737 | 410 |  |  |
| *clp*P | - | 76511 | 78589 | 119 | 756 | 294 | 666 | 244 |
| *rpl*16 | - | 82938 | 84468 | 9 | 1123 | 399 |  |  |
| *rpl*2 | - | 86147 | 87635 | 391 | 667 | 431 |  |  |
| *ycf*2 | + | 88763 | 95299 | 759 | 63 | 5715 |  |  |
| *ndh*B | - | 96508 | 98712 | 777 | 672 | 756 |  |  |
| *trn*I-GAU | + | 104260 | 105277 | 33 | 945 | 40 |  |  |
| *trn*A-UGC | + | 105358 | 106246 | 37 | 816 | 36 |  |  |
| *ycf*1 | + | 111558 | 118616 | 1912 | 42 | 1285 | 45 | 3775 |
| *ndh*A | + | 120568 | 122679 | 553 | 1020 | 539 |  |  |
| *ndh*A | - | 134102 | 136213 | 553 | 1020 | 539 |  |  |
| *ycf*1 | - | 138165 | 145223 | 1912 | 42 | 1285 | 45 | 3775 |
| *trn*A-UGC | - | 150535 | 151423 | 37 | 816 | 36 |  |  |
| *trn*I-GAU | - | 151504 | 152521 | 33 | 945 | 40 |  |  |
| *ndh*B | + | 158069 | 160273 | 777 | 672 | 756 |  |  |
| *ycf*2 | - | 161482 | 168018 | 759 | 63 | 5715 |  |  |
| *rpl*2 | + | 169146 | 170634 | 391 | 667 | 431 |  |  |

# S3 Table. Codon Usage in the tangshen plastome.

| Codon | Amino acid | Frequency | Number |
| --- | --- | --- | --- |
| GCA | A | 15.184 | 772 |
| GCC | A | 9.323 | 474 |
| GCG | A | 6.373 | 324 |
| GCT | A | 22.953 | 1167 |
| TGC | C | 3.914 | 199 |
| TGT | C | 9.264 | 471 |
| GAC | D | 9.5 | 483 |
| GAT | D | 28.657 | 1457 |
| GAA | E | 36.21 | 1841 |
| GAG | E | 14.771 | 751 |
| TTC | F | 20.042 | 1019 |
| TTT | F | 35.226 | 1791 |
| GGA | G | 25.136 | 1278 |
| GGC | G | 8.89 | 452 |
| GGG | G | 12.371 | 629 |
| GGT | G | 19.314 | 982 |
| CAC | H | 6.471 | 329 |
| CAT | H | 16.797 | 854 |
| ATA | I | 24.113 | 1226 |
| ATC | I | 17.092 | 869 |
| ATT | I | 39.887 | 2028 |
| AAA | K | 42.248 | 2148 |
| AAG | K | 19.098 | 971 |
| CTA | L | 14.063 | 715 |
| CTC | L | 7.671 | 390 |
| CTG | L | 6.845 | 348 |
| CTT | L | 23.327 | 1186 |
| TTA | L | 30.329 | 1542 |
| TTG | L | 22.973 | 1168 |
| ATG | M | 22.363 | 1137 |
| AAC | N | 11.27 | 573 |
| AAT | N | 31.548 | 1604 |
| CCA | P | 11.801 | 600 |
| CCC | P | 7.867 | 400 |
| CCG | P | 6.943 | 353 |
| CCT | P | 14.476 | 736 |
| CAA | Q | 25.962 | 1320 |
| CAG | Q | 8.693 | 442 |
| AGA | R | 19.806 | 1007 |
| AGG | R | 7.946 | 404 |
| CGA | R | 13.866 | 705 |
| CGC | R | 4.838 | 246 |
| CGG | R | 5.979 | 304 |
| CGT | R | 10.955 | 557 |
| AGC | S | 6.353 | 323 |
| AGT | S | 14.928 | 759 |
| TCA | S | 14.338 | 729 |
| TCC | S | 11.349 | 577 |
| TCG | S | 7.415 | 377 |
| TCT | S | 21.124 | 1074 |
| ACA | T | 14.358 | 730 |
| ACC | T | 8.713 | 443 |
| ACG | T | 6.609 | 336 |
| ACT | T | 20.357 | 1035 |
| GTA | V | 17.643 | 897 |
| GTC | V | 7.985 | 406 |
| GTG | V | 7.808 | 397 |
| GTT | V | 18.941 | 963 |
| TGG | W | 17.898 | 910 |
| TAC | Y | 7.533 | 383 |
| TAT | Y | 29.07 | 1478 |
| TAA | * | 6.176 | 314 |
| TAG | * | 4.661 | 237 |
| TGA | * | 4.386 | 223 |

# S4 Table. Microsatellite repeats in the tangshen plastome. The structure of Microsatellite repeats is presented as repeat units surrounded by parenthesis and the numbers of repeat units.

| Type | Structure of Microsatellite Repeats | Size (bp) | Start | End | Region (Gene Names) |
| --- | --- | --- | --- | --- | --- |
| p2 | (TA)7 | 14 | 32314 | 32327 | *rpo*C1 |
| p1 | (A)14 | 14 | 34718 | 34731 | *rpo*C2 |
| c | (T)10g(A)12 | 23 | 38024 | 38046 | IGS (*rpo*C2- *rps*2) |
| p1 | (A)14 | 14 | 39769 | 39782 | IGS (*atp*I- *rps*12) |
| p2 | (AT)14 | 28 | 43179 | 43206 | IGS (*psa*J- *trn*P-UGG) |
| c | (AT)8a(AT)8 | 33 | 46466 | 46498 | IGS (*psb*J- *pet*A) |
| p1 | (T)10 | 10 | 48990 | 48999 | *cem*A |
| c | (TA)8t(TA)8 | 33 | 49577 | 49609 | IGS (*cem*A- *ycf*4) |
| p1 | (A)10 | 10 | 50787 | 50796 | IGS (*ycf*4- *psa*I) |
| p1 | (A)10 | 10 | 66058 | 66067 | IGS (*trn*M-CAU- *trn*T-UGU) |
| p2 | (AT)7 | 14 | 68770 | 68783 | IGS (*trn*Q-UUG- *psb*K) |
| p1 | (T)12 | 12 | 69505 | 69516 | IGS (*psb*K- *psb*I) |
| p3 | (TTC)14 | 42 | 78506 | 78547 | *clp*P |
| p3 | (GAG)8 | 24 | 112536 | 112559 | *ycf*1 |
| p1 | (A)10 | 10 | 112936 | 112945 | *ycf*1 |
| p1 | (A)10 | 10 | 116068 | 116077 | *ycf*1 |
| p1 | (A)10 | 10 | 118030 | 118039 | *ycf*1 |
| p1 | (A)12 | 12 | 121722 | 121733 | *ndh*A |
| p1 | (T)11 | 11 | 127303 | 127313 | IGS (*ndh*F- *rpl*32) |
| p1 | (T)10 | 10 | 127831 | 127840 | IGS (*ndh*F- *trn*L-UAG) |
| p1 | (A)12 | 12 | 128027 | 128038 | IGS (*rpl*32- *trn*L-UAG) |
| p2 | (AT)6 | 12 | 131884 | 131895 | IGS (*psa*C- *ndh*E) |
| p1 | (T)12 | 12 | 135048 | 135059 | *ndh*A |
| p1 | (T)10 | 10 | 138742 | 138751 | *ycf*1 |
| p1 | (T)10 | 10 | 140704 | 140713 | *ycf*1 |
| p1 | (T)10 | 10 | 143836 | 143845 | *ycf*1 |
| p3 | (TCC)8 | 24 | 144220 | 144243 | *ycf*1 |

p: palindromic; IGS: intergenic spacer.

# S5 Table. Tandem repeats in the tangshen plastome.

| Start and End Sites of the Tandem Repeats | Period  Size（bp） | Copy Number | Consensus Size  （bp） | Percent Matches | %  Indels | Score | Bases number  A G C T | | | | Extropy （0-2） |
| --- | --- | --- | --- | --- | --- | --- | --- | --- | --- | --- | --- |
| 5222-5309 | 39 | 2.3 | 39 | 92 | 7 | 144 | 50 | 3 | 4 | 42 | 1.39 |
| 5949-6023 | 36 | 2.1 | 36 | 100 | 0 | 150 | 44 | 10 | 25 | 20 | 1.83 |
| 6467-6516 | 25 | 2 | 25 | 96 | 0 | 91 | 50 | 8 | 10 | 32 | 1.65 |
| 46452-46498 | 17 | 2.8 | 17 | 93 | 0 | 76 | 46 | 2 | 0 | 51 | 1.13 |
| 49576-49611 | 17 | 2.1 | 17 | 100 | 0 | 72 | 44 | 0 | 0 | 55 | 0.99 |
| 49579-49608 | 15 | 2 | 15 | 100 | 0 | 60 | 46 | 0 | 0 | 53 | 1 |
| 81398-81588 | 81 | 2.4 | 81 | 98 | 0 | 366 | 48 | 6 | 9 | 34 | 1.63 |
| 87995-88092 | 36 | 2.7 | 36 | 95 | 0 | 169 | 46 | 9 | 11 | 32 | 1.71 |
| 88037-88177 | 57 | 2.5 | 57 | 96 | 0 | 264 | 46 | 11 | 10 | 31 | 1.74 |
| 89336-89650 | 63 | 5 | 63 | 98 | 0 | 603 | 32 | 14 | 22 | 31 | 1.94 |
| 91555-91656 | 48 | 2.1 | 48 | 98 | 0 | 195 | 39 | 18 | 14 | 27 | 1.9 |
| 91873-91932 | 30 | 2 | 30 | 90 | 0 | 93 | 43 | 20 | 8 | 28 | 1.8 |
| 100781-100815 | 18 | 1.9 | 18 | 100 | 0 | 70 | 20 | 17 | 17 | 45 | 1.85 |
| 105290-105320 | 8 | 3.9 | 8 | 95 | 0 | 53 | 74 | 0 | 16 | 9 | 1.07 |
| 113423-113522 | 42 | 2.4 | 42 | 91 | 0 | 164 | 49 | 10 | 13 | 28 | 1.73 |
| 114020-114258 | 63 | 3.7 | 63 | 94 | 5 | 397 | 45 | 14 | 25 | 15 | 1.84 |
| 114494-114712 | 42 | 5.2 | 42 | 94 | 0 | 375 | 39 | 7 | 26 | 26 | 1.82 |
| 114768-115080 | 63 | 5 | 63 | 90 | 0 | 329 | 53 | 10 | 34 | 1 | 1.45 |
| 117150-117210 | 30 | 2 | 30 | 96 | 0 | 113 | 52 | 8 | 13 | 26 | 1.67 |
| 117627-117693 | 33 | 2 | 33 | 91 | 0 | 107 | 53 | 1 | 23 | 20 | 1.54 |
| 117682-117821 | 21 | 6.7 | 21 | 99 | 0 | 271 | 48 | 8 | 22 | 20 | 1.76 |
| 117661-117821 | 42 | 3.8 | 42 | 95 | 1 | 270 | 49 | 8 | 23 | 19 | 1.75 |
| 117661-117821 | 63 | 2.6 | 63 | 93 | 2 | 279 | 49 | 8 | 23 | 19 | 1.75 |
| 119164-119282 | 57 | 2.1 | 56 | 95 | 1 | 211 | 53 | 10 | 16 | 19 | 1.71 |
| 137499-137617 | 57 | 2.1 | 56 | 95 | 1 | 211 | 19 | 16 | 10 | 53 | 1.71 |
| 138960-139120 | 63 | 2.5 | 62 | 92 | 4 | 268 | 19 | 23 | 8 | 49 | 1.75 |
| 138960-139099 | 21 | 6.7 | 21 | 99 | 0 | 271 | 20 | 22 | 8 | 48 | 1.76 |
| 139088-139154 | 33 | 2 | 33 | 91 | 0 | 107 | 20 | 23 | 1 | 53 | 1.54 |
| 139571-139631 | 30 | 2 | 30 | 96 | 0 | 113 | 26 | 13 | 8 | 52 | 1.67 |
| 141701-142013 | 63 | 5 | 63 | 90 | 0 | 347 | 1 | 34 | 10 | 53 | 1.45 |
| 142069-142287 | 42 | 5.2 | 42 | 94 | 0 | 375 | 26 | 26 | 7 | 39 | 1.82 |
| 142561-142789 | 36 | 6.6 | 36 | 90 | 8 | 386 | 15 | 24 | 14 | 45 | 1.84 |
| 142523-142761 | 63 | 3.7 | 63 | 90 | 9 | 397 | 15 | 25 | 14 | 45 | 1.84 |
| 143265-143358 | 42 | 2.2 | 42 | 94 | 0 | 161 | 28 | 13 | 10 | 46 | 1.77 |
| 151461-151491 | 8 | 3.9 | 8 | 95 | 0 | 53 | 9 | 16 | 0 | 74 | 1.07 |
| 155966-156000 | 18 | 1.9 | 18 | 100 | 0 | 70 | 45 | 17 | 17 | 20 | 1.85 |
| 164239-164284 | 21 | 2.2 | 21 | 92 | 0 | 74 | 30 | 8 | 34 | 26 | 1.86 |
| 165125-165226 | 48 | 2.1 | 48 | 98 | 0 | 195 | 27 | 14 | 18 | 39 | 1.9 |
| 167131-167445 | 63 | 5 | 63 | 98 | 0 | 603 | 31 | 22 | 14 | 32 | 1.94 |
| 168689-168786 | 36 | 2.7 | 36 | 95 | 0 | 169 | 32 | 11 | 9 | 46 | 1.71 |
| 168604-168744 | 57 | 2.5 | 57 | 96 | 0 | 264 | 31 | 10 | 11 | 46 | 1.74 |

# S6 Table. Dispersed repeats in the tangshen plastome.

| The repeat length of the first part | The starting site of the first part | Matching direction | The repeat length of the second part | The starting site of the second part | interval distance of repeats | E-value |
| --- | --- | --- | --- | --- | --- | --- |
| 540 | 26852 | D | 540 | 110876 | -3 | 0.00E+00 |
| 540 | 26852 | P | 540 | 145364 | -3 | 0.00E+00 |
| 349 | 26619 | P | 349 | 87662 | -2 | 3.40E-195 |
| 349 | 26619 | D | 349 | 168769 | -2 | 3.40E-195 |
| 333 | 26635 | P | 333 | 87662 | -1 | 2.67E-188 |
| 333 | 26635 | D | 333 | 168785 | -1 | 2.67E-188 |
| 285 | 37564 | P | 285 | 75895 | -3 | 2.19E-154 |
| 278 | 37538 | P | 278 | 75928 | -3 | 3.32E-150 |
| 270 | 36754 | P | 270 | 76478 | -3 | 1.99E-145 |
| 252 | 89335 | D | 252 | 89398 | -3 | 1.11E-134 |
| 252 | 89335 | P | 252 | 167130 | -3 | 1.11E-134 |
| 252 | 89398 | P | 252 | 167193 | -3 | 1.11E-134 |
| 252 | 167130 | D | 252 | 167193 | -3 | 1.11E-134 |
| 242 | 66060 | D | 242 | 77785 | -3 | 1.03E-128 |
| 236 | 27156 | D | 236 | 111180 | -2 | 1.68E-127 |
| 236 | 27156 | P | 236 | 145364 | -2 | 1.68E-127 |
| 234 | 66068 | D | 234 | 77793 | -2 | 2.64E-126 |
| 236 | 6126 | D | 236 | 67045 | -3 | 3.92E-125 |
| 226 | 36798 | P | 226 | 76478 | -1 | 4.78E-124 |
| 223 | 89364 | D | 223 | 89427 | -2 | 1.00E-119 |
| 223 | 89364 | P | 223 | 167130 | -2 | 1.00E-119 |
| 223 | 89427 | P | 223 | 167193 | -2 | 1.00E-119 |
| 211 | 89376 | D | 211 | 89439 | -1 | 4.79E-115 |
| 211 | 89376 | P | 211 | 167130 | -1 | 4.79E-115 |
| 211 | 89439 | P | 211 | 167193 | -1 | 4.79E-115 |
| 208 | 66094 | D | 208 | 77819 | -1 | 3.02E-113 |
| 207 | 21630 | D | 207 | 65175 | -2 | 3.72E-110 |
| 187 | 84477 | P | 187 | 88178 | -1 | 1.19E-100 |
| 187 | 84477 | D | 187 | 168415 | -1 | 1.19E-100 |
| 177 | 26791 | P | 177 | 87662 | 0 | 2.23E-97 |
| 177 | 26791 | D | 177 | 168941 | 0 | 2.23E-97 |
| 188 | 37262 | P | 188 | 76299 | -3 | 1.57E-96 |
| 184 | 37499 | P | 184 | 76061 | -3 | 3.76E-94 |
| 166 | 167216 | D | 166 | 167279 | -2 | 1.15E-85 |
| 165 | 37685 | P | 165 | 75896 | -2 | 4.56E-85 |
| 154 | 36870 | P | 154 | 76478 | 0 | 1.57E-83 |
| 166 | 89335 | D | 166 | 89461 | -3 | 1.89E-83 |
| 166 | 89335 | P | 166 | 167153 | -3 | 1.89E-83 |
| 166 | 89461 | P | 166 | 167279 | -3 | 1.89E-83 |
| 166 | 167153 | D | 166 | 167279 | -3 | 1.89E-83 |
| 160 | 89364 | D | 160 | 89490 | -3 | 6.94E-80 |
| 160 | 89364 | P | 160 | 167130 | -3 | 6.94E-80 |
| 160 | 89490 | P | 160 | 167256 | -3 | 6.94E-80 |
| 160 | 167130 | D | 160 | 167256 | -3 | 6.94E-80 |
| 146 | 27246 | D | 146 | 111270 | -1 | 4.51E-76 |
| 146 | 27246 | P | 146 | 145364 | -1 | 4.51E-76 |
| 148 | 89376 | D | 148 | 89502 | -2 | 6.30E-75 |
| 148 | 89376 | P | 148 | 167130 | -2 | 6.30E-75 |
| 148 | 89502 | P | 148 | 167256 | -2 | 6.30E-75 |

D: direct repeat, forward repeat sequence; P: palindromic repeat, palindromic repeat sequence.

# S7 Table. The five pairs of primers for the amplification of DNA barcodes.

| Primer ID | Sequence | Product length (bp) |
| --- | --- | --- |
| Com1_F | GTCATTATCCCTCGAGAAGTAG | 320 |
| Com1_R | GCTGACCTGCTAACCTCTATAC |  |
| Com2_F | CTCTTACTGTAACCGGTTTGTC | 270 |
| Com2_R | GTCCGGTTCTGTAGTAGAGATG |  |
| Com3_F | CGTGTGATTAGTCTAGGGTTTC | 260 |
| Com3_R | GCCCTAGTCCTAATAGATTGAC |  |
| Com4_F | TAGAAGGTGGGTTGAAAGGAGT | 364-370 |
| Com4_R | AGCCTACTCTCAAAATCGAACC |  |
| Com5_F | CTTCTATGCGAAAGTGTTCC | 325 |
| Com5_R | AGTTTGACTCCCAAAGAAGC |  |


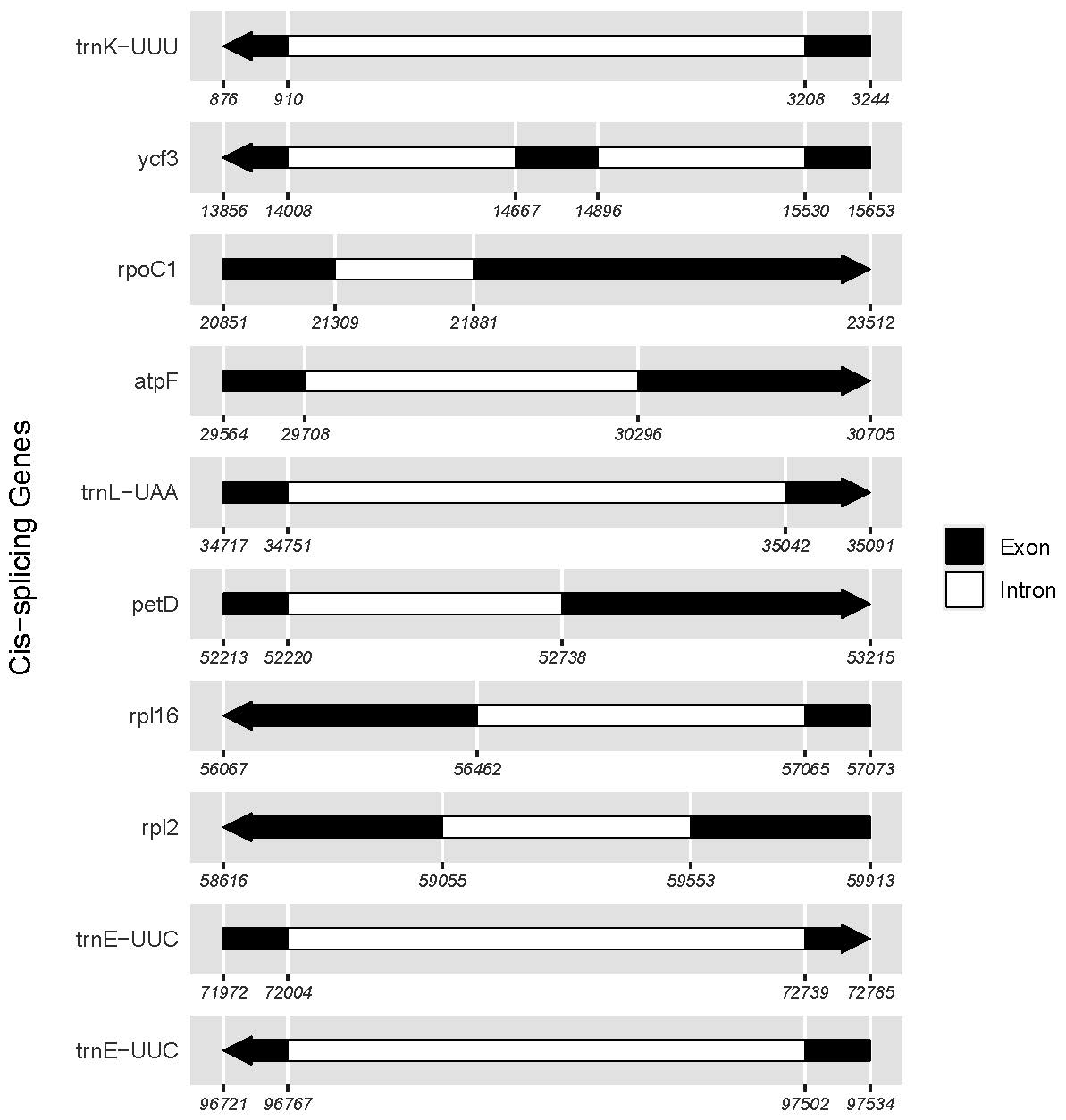


# S1 Fig. The cis-splicing genes of the tangshen plastome were created by CPGview-RSG. The column on the left is the name of the gene. The black and white regions represent the exon and intron, respectively. The numbers below the arrow of each gene represent the start and end sites of the exon and intron.


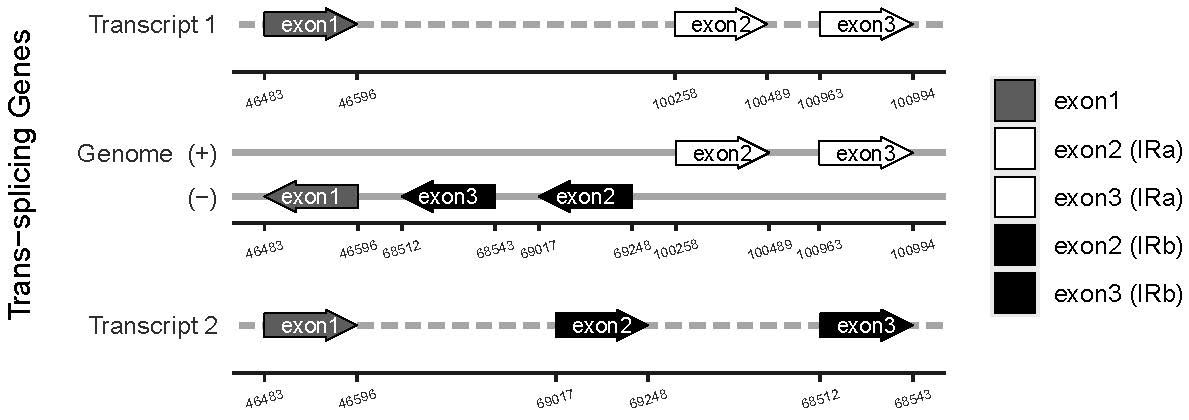


# S2 Fig. The trans-splicing of *rps*12 gene in the tangshen plastome created by CPGview-RSG. Different colors represent exons in different regions, as shown on the right. Exon1 is in the single-copy region. Exon2 (IRa) and exon3 (IRa) are in the IRa region. And exon2 (IRb) and exon3 (IRb) are in the IRb region. Exon1, exon2 (IRa), and exon3 (IRa) are linked together to form an *rps*12 gene. Exon1, exon2 (IRb), and exon3 (IRb) linked together to form another *rps*12 gene. Transcripts 1 and 2 represent the products of two copies of the *rps*12 gene. The numbers below the arrow of each exon represent the start and end sites.


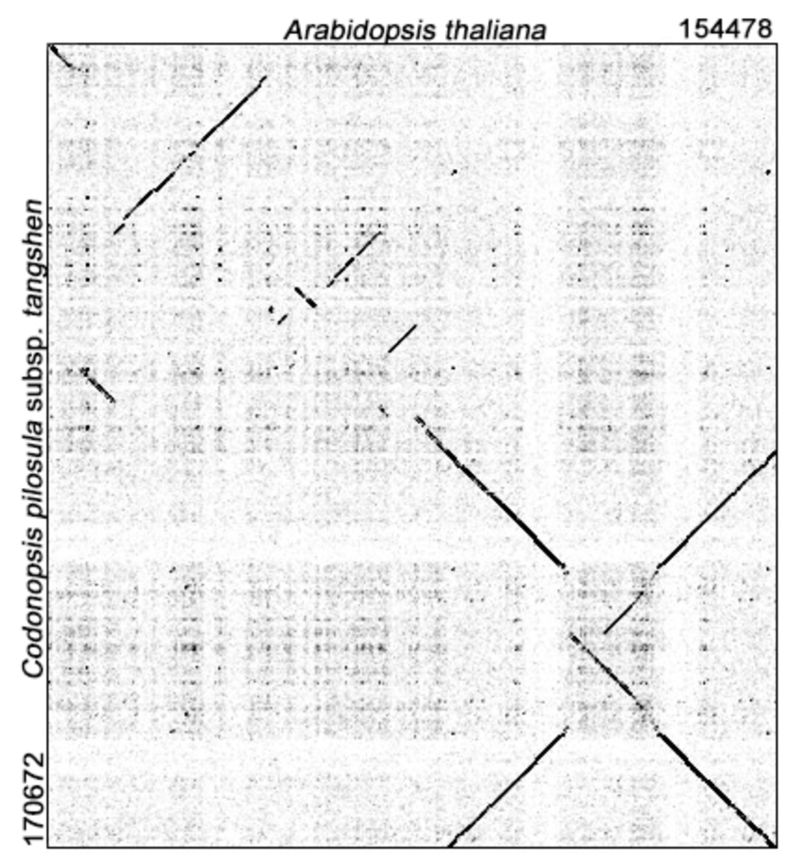


# S3 Fig. Dotplot of *Arabidopsis thaliana* and tangshen plastomes. The horizontal direction is the *A. thaliana* plastome. The vertical direction is the tangshen plastome.


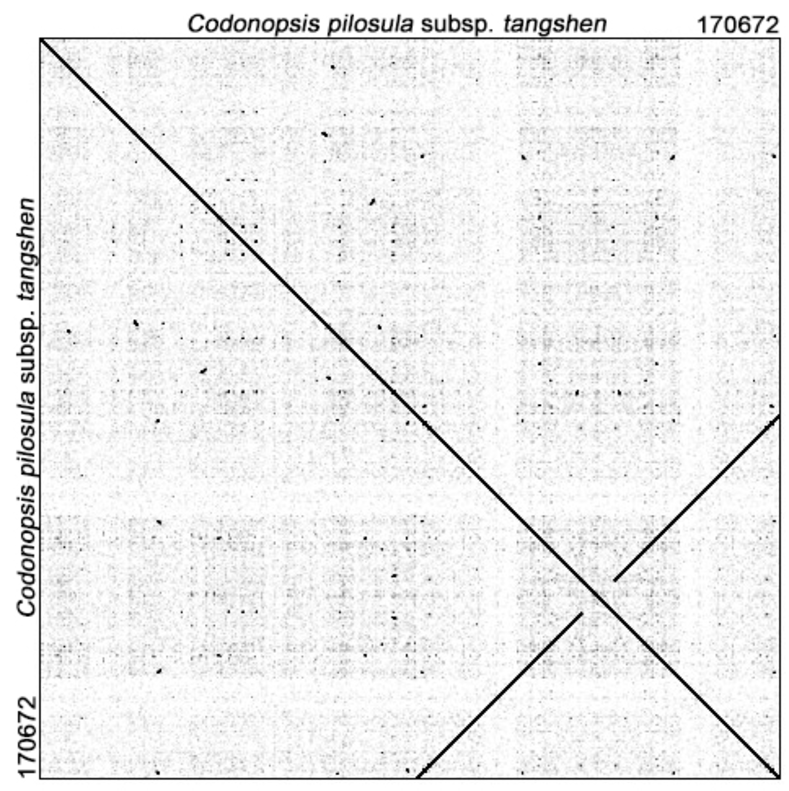


# S4 Fig. Dotplot of tangshen plastome vs itself. The horizontal direction and the vertical direction are all the tangshen plastome.


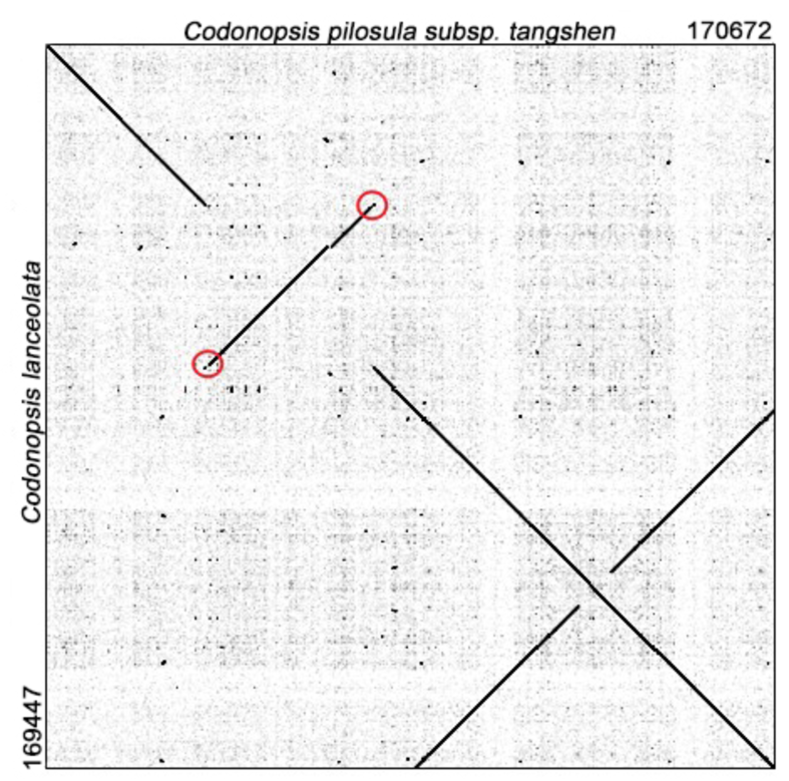


# S5 Fig. Dotplot of tangshen and *C. lanceolata* plastomes. The horizontal and vertical directions show the tangshen and *C. lanceolata* plastomes. The red circles indicate the repeat sequences at both ends of the inversion region in tangshen.


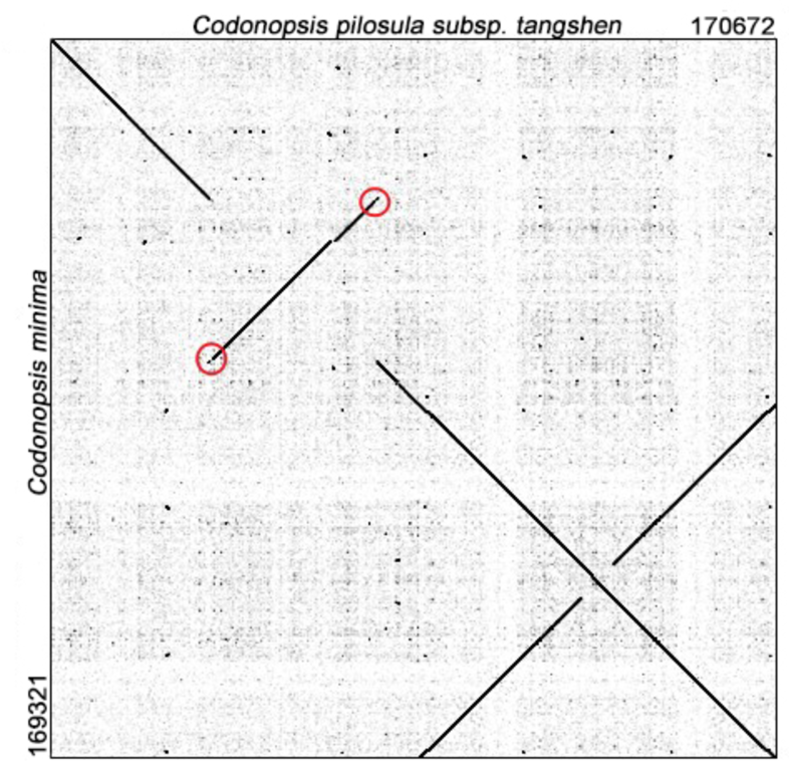


# S6 Fig. Dotplot of tangshen and *C. minima* plastomes. The horizontal and vertical directions show the tangshen and *C. minima* plastomes. The red circles indicate the repeat sequences at both ends of the inversion region in tangshen.


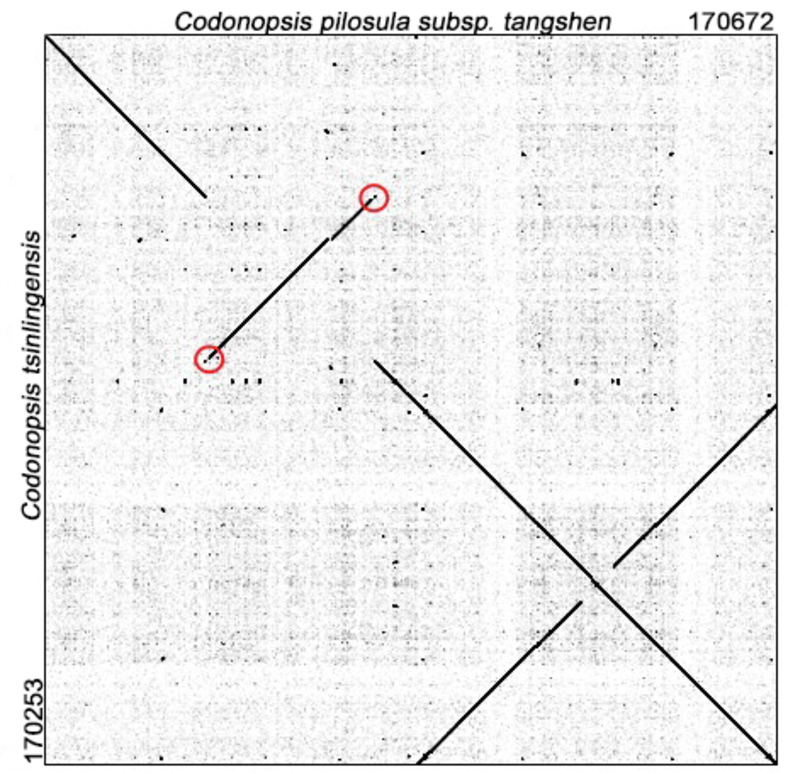


# S7 Fig. Dotplot of tangshen and *C. tsinglingensis* plastomes. The horizontal and vertical directions show the tangshen and *C. tsinglingensis* plastomes. The red circles indicate the repeat sequences at both ends of the inversion region in tangshen.


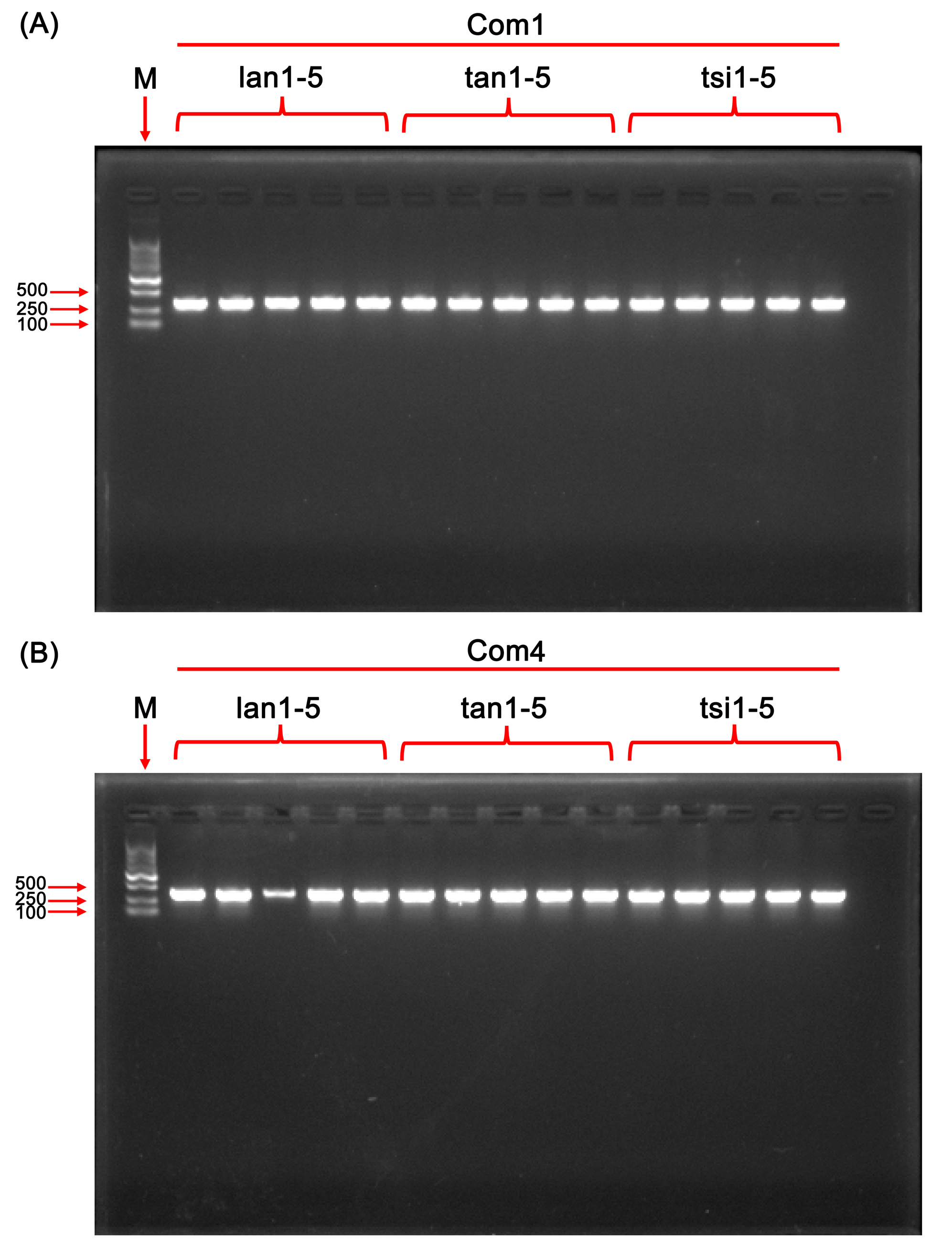


# S8 Fig. The full-length gel electrophoresis results from amplifying molecular markers Com1 (A) and Com4 (B). Lane M was the marker of DL2000. The lanes from left to right correspond to products amplified from the individual 1 to 5 of *Codonopsis lanceolata* (lan), *Codonopsis pilosula* subsp. *tangshen* (tan) and *Codonopsis tsilingensis* (tsi) by primer Com1 and Com4, respectively.


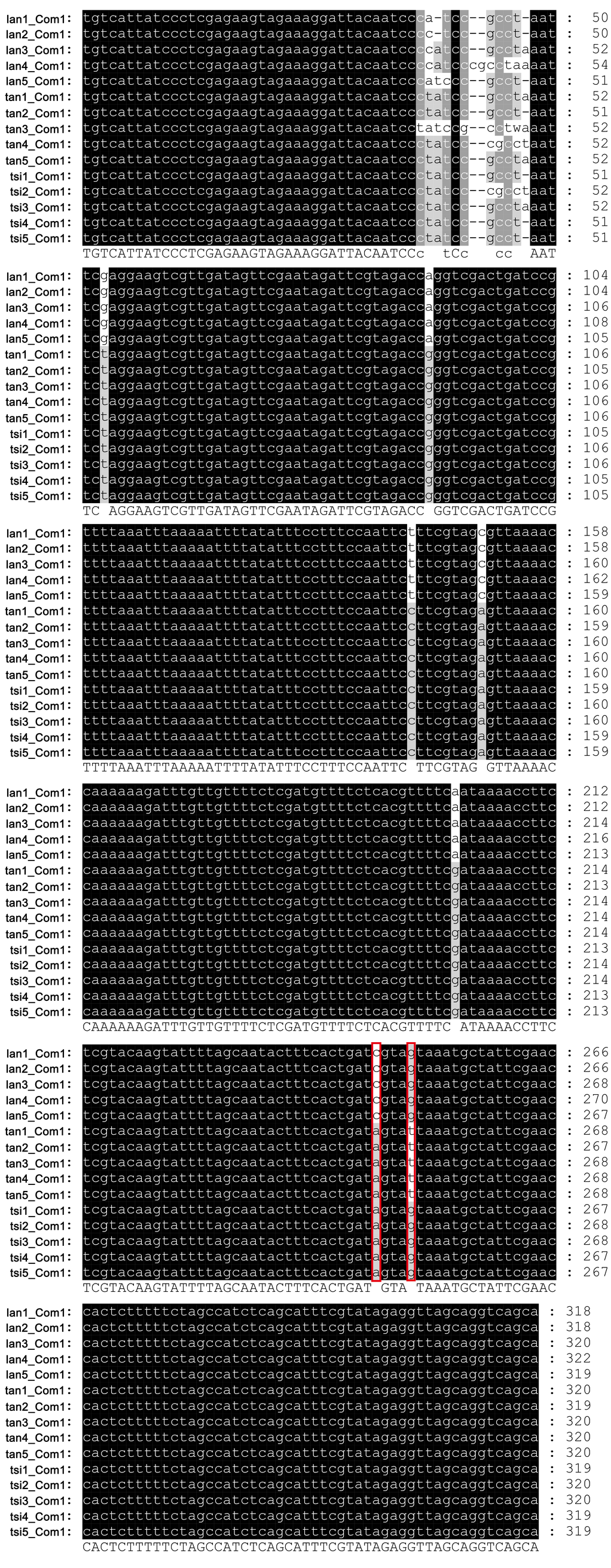


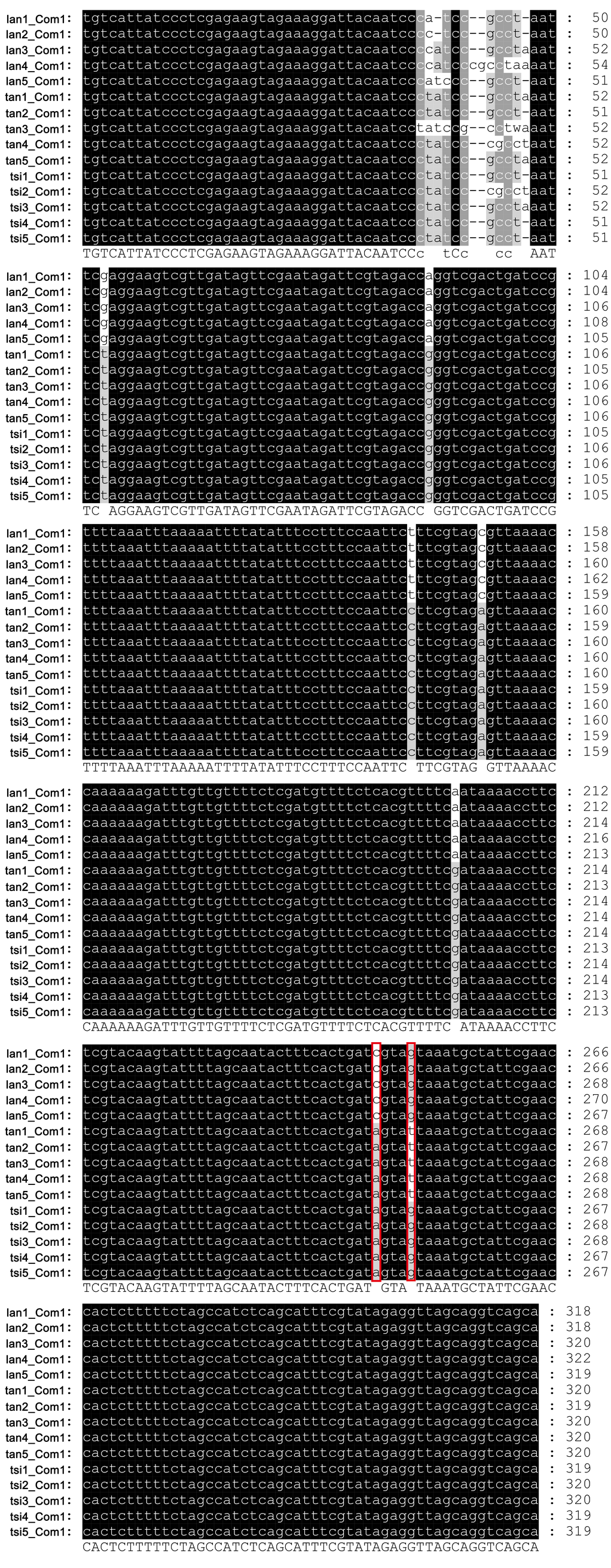


# S9 Fig. The alignment of Sanger sequencing results of the PCR products amplified by Com1 primers. The ID of each sequence is shown on the left side of each panel. The ID includes the abbreviation of the species name, individual plant id, and primer name. The rightmost number in each column represents the position of the base at that position in the amplicon. The SNPs which can distinguish these three species are shown in red squares. The nucleotides identical across all plastomes are shaded in black. Those conserved in 60% of the sequences are shaded in gray. Lan: *Codonopsis lanceolata*; tan: *Codonopsis pilosula* subsp. *tangshen*; tsi: *Codonopsis tsinlingensis*. Arabic numerals represent different individuals.


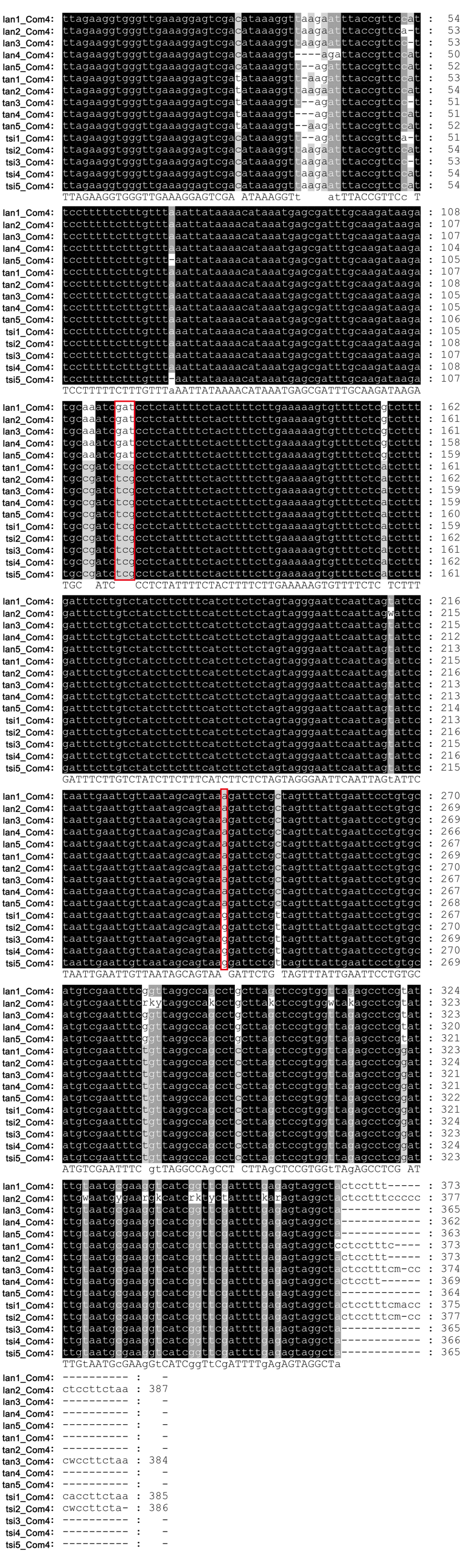


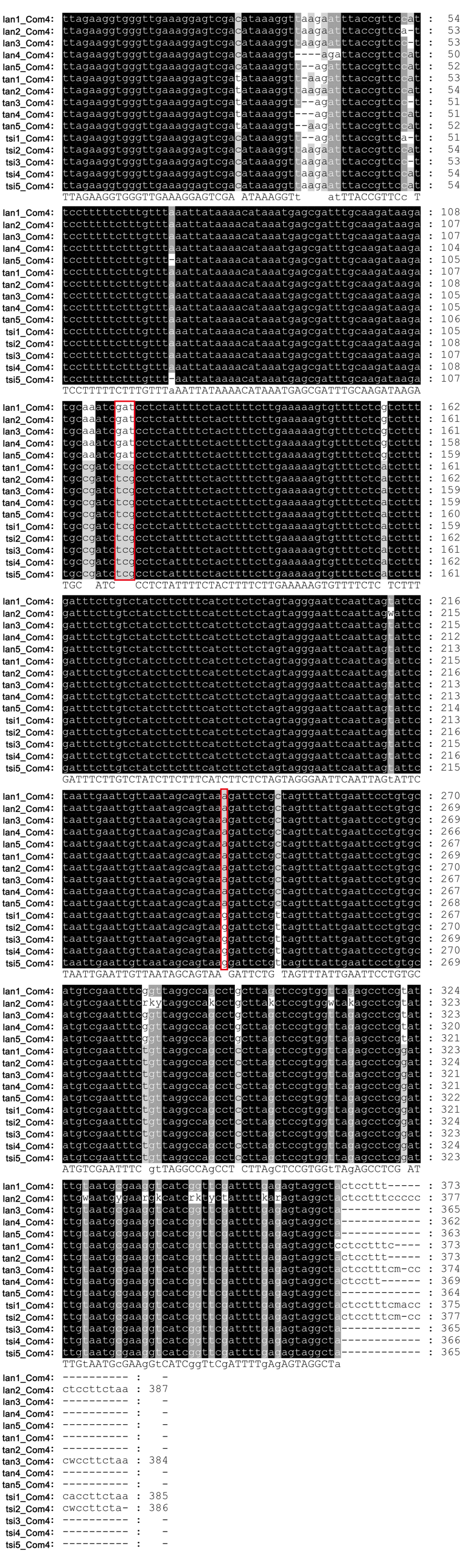


# S10 Fig. The alignment of Sanger sequencing results of the PCR products amplified by Com4 primers. The ID of each sequence is shown on the left side of each panel. The ID includes the abbreviation of the species name, individual plant id, and primer name. The rightmost number in each column represents the position of the base at that position in the amplicon. The SNPs and Indel, which can distinguish these three species, are shown in red squares. The nucleotides identical across all plastomes are shaded in black. Those conserved in 60% of the sequences are shaded in gray. Lan: *Codonopsis lanceolata*; tan: *Codonopsis pilosula* subsp. *tangshen*; tsi: *Codonopsis tsinlingensis*. Arabic numerals represent different individuals.


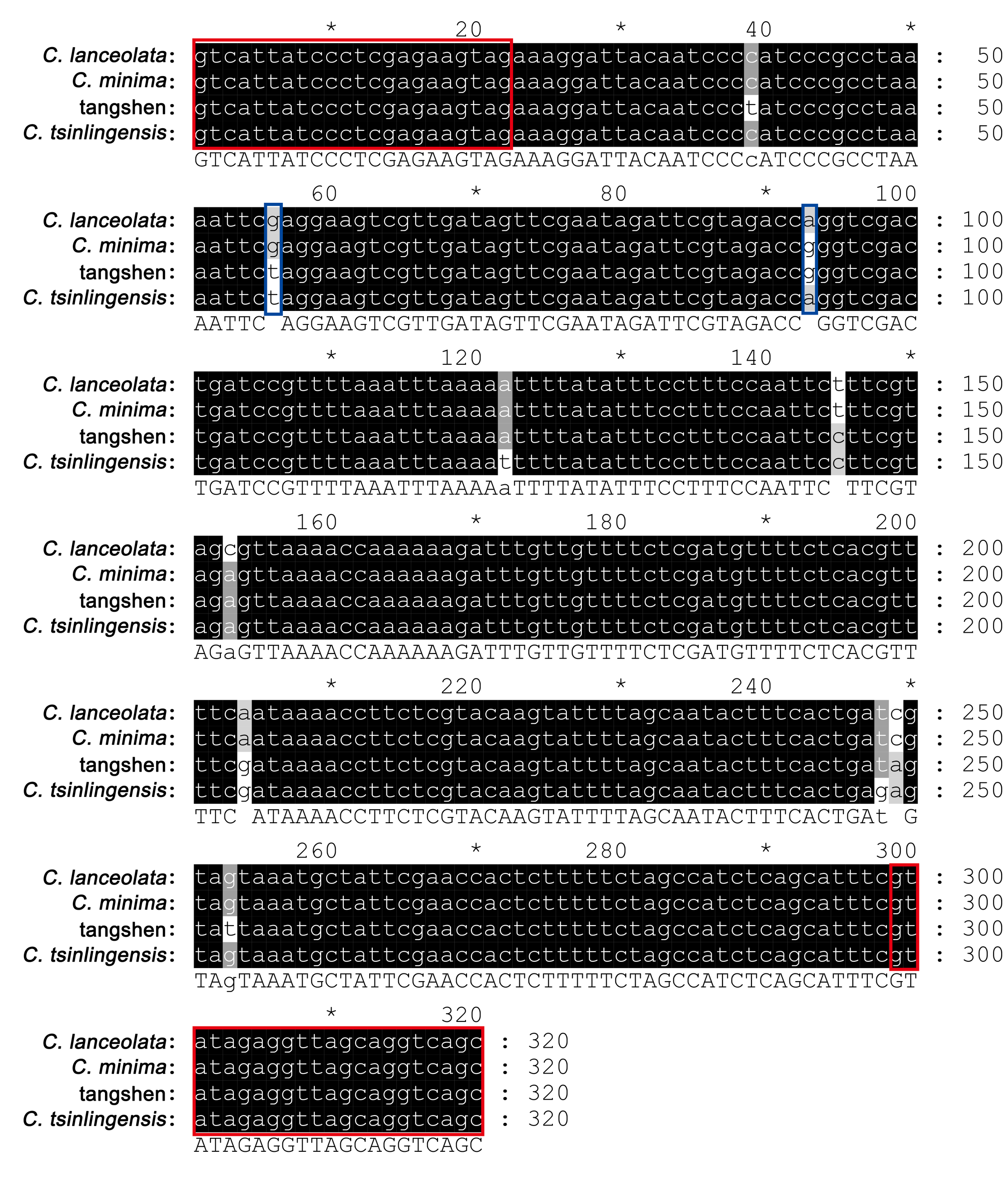


# S11 Fig. The alignment of amplicons in four *Codonopsis* plastomes produced by Com1 primers. The ID of each sequence is shown on the left side of each panel. The ID is the species' name. The forward and reverse primers for amplification are shown in the red squares. The SNPs which can distinguish these four species are shown in blue squares. The nucleotides identical across all plastomes are shaded in black. Those conserved in 60% of the sequences are shaded in gray. Lan: *Codonopsis lanceolata*; tan: *Codonopsis pilosula* subsp. *tangshen*; tsi: *Codonopsis tsinlingensis*.


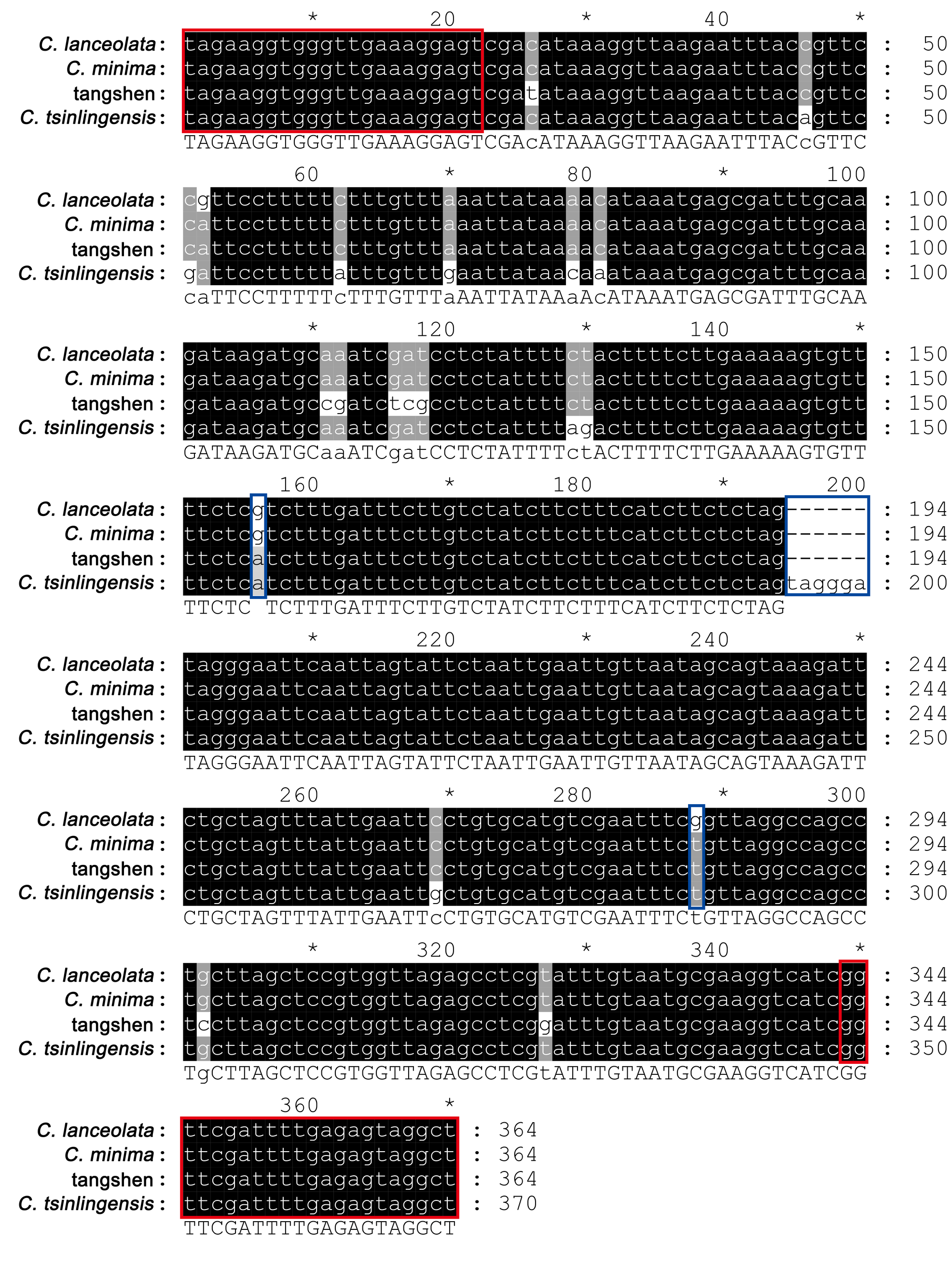


# S12 Fig. The alignment of amplicons in four *Codonopsis* plastomes produced by Com4 primers. The ID of each sequence is shown on the left side of each panel. The ID is the species' name. The forward and reverse primers for amplification are shown in the red squares. The SNPs and Indel, which can distinguish these four species, are shown in blue squares. The nucleotides identical across all plastomes are shaded in black. Those conserved in 60% of the sequences are shaded in gray. Lan: *Codonopsis lanceolata*; tan: *Codonopsis pilosula* subsp. *tangshen*; tsi: *Codonopsis tsinlingensis*.
